# Revision of the Genus *Cyanoboletus* (*Boletaceae*) in the Mediterranean Basin with Notes on Arsenic Hyperaccumulation

**DOI:** 10.3390/jof12050315

**Published:** 2026-04-25

**Authors:** Alona Yu. Biketova, Isaac Garrido-Benavent, Vasco Fachada, Giampaolo Simonini, Matteo Gelardi, Boris Assyov, Elias Polemis, Antoni Conca, Roseina Woods, Georgios I. Zervakis, Jan Borovička, Andrea C. Rinaldi

**Affiliations:** 1 Jodrell Laboratory, Royal Botanic Gardens, Kew, Richmond TW9 3DS, UK; alyona.biketova@gmail.com (A.Y.B.); timal80@yahoo.it (M.G.); r.woods@kew.org (R.W.); 2 Association for Wild Fungi of Israel, P.O. Box 164, Pardesiya 42815, Israel; 3 Institute of Evolution, University of Haifa, Aba Khoushi Ave. 199, Mt. Carmel, Haifa 3498838, Israel; 4 Department of Botany and Geology, University of Valencia, Av. Vicent Andrés Estellés 19, Burjassot, E-46100 Valencia, Spain; isaac.garrido@uv.es; 5 Research Centre in Biodiversity and Genetic Resources, University of Porto, Vairão, 4485-661 Vila do Conde, Portugal; 6 Independent Researcher, Via Bellaria 8, I-42121 Reggio Emilia, Italy; giamsim@tin.it; 7 Institute of Biodiversity and Ecosystem Research, Bulgarian Academy of Sciences, 2 Gagarin Str., 1113 Sofia, Bulgaria; contact@boletales.com; 8 Laboratory of General and Agricultural Microbiology, Agricultural University of Athens, Iera Odos 75, 11855 Athens, Greece; eliasp@ath.forthnet.gr (E.P.); zervakis@aua.gr (G.I.Z.); 9 Independent Researcher, P. Poeta Joan Vimbodí 5, Ontinyent, E-46870 Valencia, Spain; tconca@gmail.com; 10 Institute of Geology of the Czech Academy of Sciences, Rozvojová 269, CZ-16500 Prague 6, Czech Republic; 11 Department of Biomedical Sciences, University of Cagliari, I-09042 Monserrato, Italy

**Keywords:** *Boletales*, taxonomy, molecular phylogeny, macrofungi biogeography, Israel, Southern Europe, *Suillelloideae*

## Abstract

This study revises the genus *Cyanoboletus* (*Boletaceae*) in the Mediterranean Basin, integrating single-locus and multi-locus phylogenetic analyses (ITS, LSU, *tef1-α*, and *rpb2*), morphological characterisation, ecology, and arsenic accumulation in basidiomes. Morphological descriptions (including a new form, *Cyanoboletus mediterraneensis* f. *pallidus*), comprehensive sampling, type studies, biogeography, macro- and microphotographs, an identification key, and a historical overview of the nomenclatural issues surrounding *C. pulverulentus*, *C. poikilochromus*, and *C. mediterraneensis* are given. An epitype collection is designated for *C. pulverulentus*. A new method to measure spore suprahilar depression has been proposed, which allowed more clear morphological separation between *C. mediterraneensis* and *C. pulverulentus*. This method may prove useful for species delimitation in other fungal groups that have asymmetric basidiospores. Additionally, we generated a new ITS sequence of the *C. sinopulverulentus* holotype and inferred its conspecificity with the later described *C. flavocontextus*. Furthermore, notes on the taxonomy of *Boletus gabretae* are presented, and its placement in the genus *Neoboletus* is suggested. *Cyanoboletus* is confirmed as a strongly supported generic clade encompassing 21 monophyletic species-level clades, 14 of which represent known species, and seven are undescribed taxa. The synonymy of *Cupreoboletus* with *Cyanoboletus* is also verified. This publication provides the tools to delimit *Cyanoboletus* species that have important conservation value, which can be used by conservationists, ecologists, and citizen scientists. It also highlights species-specific arsenic hyperaccumulation in *C. pulverulentus*, contributing to a better understanding of fungal metal uptake. Our study indicates that within *Cyanoboletus*, only *C. pulverulentus* demonstrates this characteristic and is the only known member of *Boletales* that possesses a high arsenic accumulation ability.

## 1. Introduction

Singer divided the genus *Boletus* into seven sections mainly based on morphological features such as the colour of the hymenophore, the context discolouration upon exposure and the taste of basidiomes [1]. *Boletus pulverulentus* Opat., the type species of the recently established genus *Cyanoboletus* Gelardi, Vizzini & Simonini, was placed in section *Subpruinosi* Fr. emend. Singer (type species *B. barlae* Fr. = *Hortiboletus rubellus* (Krombh.) Simonini, Vizzini & Gelardi), which is characterised by a xerocomoid habit, a yellowish context, a mild taste, rounded to angular yellow pores, and tissues that turn blue when injured or handled. Later, Lavorato and Simonini excluded species of *Xerocomus* s. l. (e.g., *H. rubellus*) from this section [2].

Gelardi and coauthors conducted the first phylogenetic analysis focused on sect. *Subpruinosi* and inferred three distinct lineages at the species level; they also described a new species, *B. sinopulverulentus* Gelardi & Vizzini, from China [3]. Wu et al., in their multi-locus (LSU, *tef1-α*, *rpb1*, and *rpb2*) megaphylogeny of the family *Boletaceae*, placed *B. pulverulentus* within the “Pulveroboletus group”, one of seven major clades at the subfamily level recognised in their study [4]. This group was further delimited to the subfamily rank *Suillelloideae* in the recent phylogenomic work by Tremble et al. (2024) [5].

Later, *Cyanoboletus* was described as a new genus, including three species: *C. pulverulentus* (Opat.) Gelardi, Vizzini & Simonini (type species), *C. sinopulverulentus* (Gelardi & Vizzini) Gelardi, Vizzini & Simonini and *C. rainisiae* (Bessette & O.K. Mill.) Gelardi, Vizzini & Simonini [6]. The latter species was later placed in the genus *Xerocomellus*, although without an examination of the type material, and is currently known as *X. rainisiae* (Bessette & O.K. Mill.) N. Siegel, C.F. Schwarz & J.L. Frank [7].

*Cyanoboletus* is characterised by small to large basidiomes, a yellowish context, intensely blueing tissues when handled or injured, and an ectomycorrhizal (ECM) lifestyle in relationships with both deciduous and coniferous host plants. Currently, the genus includes 16 species, only three of which are known in Europe and the Asian Middle East: *C. pulverulentus*; *C. poikilochromus* (Pöder, Cetto & Zuccherelli) M. Carbone, D. Puddu & P. Alvarado; and the recently described *C. mediterraneensis* Biketova, Rinaldi & Simonini [8,9,10,11,12,13,14]. Twelve additional species are known from North America and Asia [3,14,15,16,17,18,19,20,21,22,23,24]. Recently, *Boletus gabretae* Pilát was transferred to *Cyanoboletus* based solely on the literature interpretation and without an examination of any of the specimens [23]. A list of *Cyanoboletus* species was confirmed to be members of the genus in previous phylogenetic studies, and their geographical distribution is given in Table 1.

Even the most well-known *Cyanoboletus* species are relatively uncommon across their distribution range and are included in the Red Lists and the Red Data Books of some countries and regions. *Cyanoboletus pulverulentus* is listed as an endangered (EN) species with criterion B2ab(iii) in the Red Data Book of Bulgaria [25] and as vulnerable (VU) in the Red Lists of Norway and Sweden [26], as well as in the Red Data Books of two regions in the Far East of Russia: Sakhalin and Kamchatka [27,28]. *Cyanoboletus poikilochromus* is protected at the international level and included in the IUCN Red List under the category vulnerable (VU) criterion C2a(i) due to its small population size and continuing decline [29]. Therefore, members of the *Cyanoboletus* genus have important conservation value. Other *Cyanoboletus* species, except *C. cyaneitinctus*, have single or few reported collections or records and require further study. An accurate delimitation of species and data on ecology and distribution are vital for the continued assessment of species that are of conservation concern.

The ability of fungi to accumulate trace elements in their basidiomes has been known for decades [30]. Element accumulation is often species-specific [31] and not directly influenced by the element content and/or mobility in the substrate [32]. Hyperaccumulation is the extraordinary ability to accumulate a chemical element, and fungal hyperaccumulators are commonly found at sites with background soil element levels [33]. Arsenic (As) is a toxic metalloid known to accumulate in various macrofungi, including both *Ascomycota* [34] and *Basidiomycota* [35]. Hyperaccumulation of As was reported in the *Sarcosphaera coronaria* (Jacq.) J. Schröt. complex [36,37], *Thelephora penicillata* (Pers.) Fr. [38,39], and *C. pulverulentus* [40]. The ability to accumulate As has not been analysed in other species of *Cyanoboletus*, except for a single specimen of *Cyanoboletus* sp. from the USA, which had a low As content [40]. It is assumed that other *Cyanoboletus* may possess a high As accumulation ability. Therefore, such an analysis would be important to clarify the safety of the consumption of these species.

The aims of the present study were to: (1) perform a taxonomic revision of the genus *Cyanoboletus* by examining species occurring in the Mediterrane Basin (except North Africa), including relevant type studies; (2) clarify the morphological variability, biogeography, and ecology of the target species and provide an identification key; (3) describe a new xanthoid form of *C. mediterraneensis*; (4) verify the taxonomic limits of the genus and its interspecific relationships through single-locus (ITS, LSU, *tef1-α*, and *rpb2*) and multi-locus analyses and delimit species by the Genealogical Concordance Phylogenetic Species Recognition method, and (5) investigate the ability to accumulate arsenic in basidiomes of the target *Cyanoboletus* species.

## 2. Materials and Methods

### 2.1. Collection Site and Sampling

A total of 102 collections of *C. mediterraneensis* (35), *C*. *pulverulentus* (33), *C. poikilochromus* (33), and *C. sinopulverulentus* (1) were studied. The studied specimens were collected from Bulgaria, France, Greece, Hungary, Israel, Italy, Portugal, Spain, Switzerland, the UK, and China and were deposited in ACAM, HAI (defunct), HMAS, IB, K, MCVE, PO, PRM, SOMF, TO, and VAL (fungarium VAL_Myco) (acronyms from Thiers) [41], while “AB”, “ACM”, “ACR”, “GK”, “GS”, “IGB”, “MG”, and “PAn” refer to the personal fungaria of Alona Yu. Biketova, Toni Conca, Andrea C. Rinaldi, Georgios Konstantinidis, Giampaolo Simonini, Isaac Garrido-Benavent, Matteo Gelardi, and Pierluigi Angeli, respectively. In the field, latitude, longitude, and elevation were determined with a Global Positioning System (GPS) receiver using WGS 84. Herbarium numbers are cited for all samples from which morphological features were examined.

Author citations follow the Index Fungorum, Authors of Fungal Names [42]. The epitype of *C. pulverulentus* and holotype of *Cyanoboletus mediterraneensis* f. *pallidus* f. nov. are registered in MycoBank [43]. The distribution range and data on occurrences were checked in the specialised literature, GBIF [14] and iNaturalist [44]. An asterisk (*) indicates disputed territories with partially recognised independence (not UN members). The abbreviation “GP” (genetically proven) indicates the distribution of *Cyanoboletus* species by countries based on collections, whose identification was verified using DNA barcoding and phylogenetic methods. The metadata from the majority of studied collections are given in Supplementary File S1 as follows: COUNTRY, first-order administrative division (or the Nature region [45] plus district in brackets for Israel), locality, coordinates (DMS), elevation in m (if available), habitat with putative host plants, date (dd.mm.yyyy), leg. = legitur, collection number (collector’s number or additional collection number), GenBank accession number(s) of a sequence(s) or GP if available. “Ibid.” indicates that a specimen was collected in the same administrative unit as the previous collection, with fully listed location data but not necessarily in the exact location.

### 2.2. Morphological Study

Macroscopic characteristics, macro-chemical reactions (25% NH_4_OH, 30% KOH, 10% H_2_SO_4_, 10% FeSO_4_ and Melzer’s reagent) were observed on fresh basidiomes. For some collections, macro-morphological characteristics of the specimens were also examined using a Carl Zeiss Stemi DV4 stereo microscope (Zeiss, Jena, Germany). For characterisation of the colour, three colour charts were used: RI [46], BFF [47], and OAC [48]. Basidiospores were measured directly from the hymenophore of mature basidiomes, and the average sizes were calculated for each collection and used in the description; the dimensions of the average values (spore width, length, quotient (length/width ratio)(Q), and area (A)) are given as (minimum) average ± standard deviation (maximum), and average spore volume was approximated as a rotation ellipsoid (apV = (π × L × W^2^)/6 ± standard deviation). The notation (n/m/p) indicates that measurements were made on “n” randomly selected basidiospores from “m” basidiomes of “p” collections. More than 2050 basidiospores from 46 voucher collections were studied (Supplementary Table S1). Spore length and width were independently measured using 40× and 100× objectives and statistically analysed using an isoprobability ellipse (IE) [49] and kernel density estimation (KDE) methods [50]. The data for the suprahilar depression and area were blindly acquired from basidiospores in the side view and exhibiting the most pronounced suprahilar depression, using a Plan-Apochromat 100×/1.40 Oil DIC objective on a Zeiss Axio Imager Z2 microscope (Zeiss). The analysis was carried out in ImageJ v.154p [51] by segmenting the full basidiospore and subtracting its area from its computed convex hull. The resulting value is expressed as the percentage of the convex hull occupied by concavity, i.e., suprahilar depression. All variables were tested for normality using the Shapiro–Wilk test, and statistical significance was assessed with independent sample *t*-tests (*p* = 0.05, 0.01, 0.001) and Mann–Whitney tests (*p* = 0.05, 0.01, 0.001) in pandas and SciPy v.1.16.0 [52,53]. The data visualisations were generated with Seaborn v.0.13.2 and Matplotlib v. 3.10.0 [54].

Absolute sizes are given for the other microscopic structures. The width of each basidium was measured at the widest part, and the length was measured from the apex (sterigmata excluded) to the basal septum. Radial and/or vertical sections of the pileipellis were taken midway between the centre and margin of the pileus. Metachromatic, cyanophilic and iodine reactions were tested by staining the basidiospores in brilliant cresyl blue, cotton blue and Melzer’s reagent, respectively. The basidiospores of selected collections (*C. mediterraneensis* K-M000265124, K-M000265125, K-M001443116, K-M001445227, K-M001445823 and PO-F2442; *C. pulverulentus* K-M001445690 and PO-F2601 and *C. poikilochromus* K-M000156117, K-M001441531 and MG1004) were also analysed using scanning electron microscopes Zeiss Ultra-Plus FEG-SEM HR (Zeiss) (operated at 4–5 kV) equipped with an Oxford EDS SDD detector (Oxford Instruments, Abingdon, UK) and Hitachi Regulus 8230 FE-SEM (Hitachi High-Tech Corporation, Tokyo, Japan) (operated at 1–10 kV) equipped with an Oxford Ultim Extreme detector (Oxford Instruments).

### 2.3. DNA Extraction, PCR Amplification and DNA Sequencing

Genomic DNA of the majority of specimens was isolated from dried basidiomes using the NucleoSpin Plant II kit (MACHEREY-NAGEL, Düren, Germany), with minor modifications, and the CTAB method. The amplification of marker loci was performed following standard procedures. The following primers were used: ITS1F, ITS4B, ITS2 and ITS3 for internal transcribed spacer (ITS) [55,56] and LR0R, LR5, and LR7 for nuclear large subunit ribosomal DNA (LSU) [57,58]. The cleanup of some PCR products was achieved using ExoSAP-IT™ (Applied Biosystems™, Waltham, MA, USA) and labelling with BigDye™ Terminator v3.1 Cycle Sequencing Kit (Applied Biosystems™) using the manufacturer’s protocols. Processed PCR products were sequenced using ABI PRISM^®^ 3700 XL Genetic Analyzer (Applied Biosystems™) at the University of Haifa (Israel), and Royal Botanic Gardens, Kew (UK), or generated by LGC Genomics (Berlin, Germany) and ALVALAB (Oviedo, Spain).

Sequences were assembled and edited using the Sequencher v. 5.4.6 (Gene Codes Corporation, Ann Arbor, MI, USA). By using the same AB1 files of the forward and reverse ITS sequences from the holotype of *C. sinopulverulentus* that were produced by Gelardi et al., 2013 [3], we were able to generate a higher-quality consensus sequence than previously published. All newly generated sequences were submitted to GenBank [59], and their accession numbers are listed in Supplementary Table S2.

### 2.4. Sequence Alignment, Phylogenetic Analyses, and Species Delimitation

Sequences of species in *Cyanoboletus*, either already identified or identified by us using similarity scores based on the Nucleotide Basic Local Alignment Search Tool (BLASTn in NCBI) [59,60], were obtained from the public database INSDCk. Our target loci were: the full ITS region, partial LSU (D1/D2 domains), partial translation elongation factor 1-α (*tef1*-α), and partial DNA-directed RNA polymerase II subunit 2 gene (*rpb2*; domains 5–11). The downloaded target loci sequences were restricted to complete or nearly complete (≥50% bp). The voucher number and collection country data were determined for all published sequences by consulting the information provided in the source database and relevant publications. These data are included in Supplementary Table S2. MAFFT v. 7.490 [61,62] was used to generate a multiple sequence alignment (MSA) independently for each marker with the following parameters: the FFT-NS-I x1000 algorithm, the 200PAM/k = 2 scoring matrix, a gap open penalty of 1.5 and an offset value of 0.123. The resulting alignments were manually optimised in Geneious Prime v. 2025.0.2 (a) to replace gaps at the ends of shorter sequences with an IUPAC base representing any base (“N”), (b) to trim ends of longer sequences in the ITS MSA that included part of the 18S–28S ribosomal subunits, and (c) to annotate the exon and intron regions in protein-coding markers (e.g., *tef1*-α, *rpb2*).

Phylogenetic reconstructions were performed using the maximum likelihood (ML) and Bayesian inference (BI) methods. The online version of RAxML v. 8.2.12, hosted at the CIPRES Science Gateway [63,64], was used to estimate a multi-locus phylogeny under an ML framework. Species in the genus *Lanmaoa* G. Wu & Zhu L. Yang and *Rugiboletus* G. Wu & Zhu L. Yang were included as outgroups. Prior to concatenation, and to test for topological incongruence among sequence datasets, we inferred ML trees independently for each locus with RAxML, using 1000 bootstrap pseudoreplicates, and assumed bootstrap values of ≥70% as significant for the conflicting relationships among the same set of taxa [65]. Because no conflicts were detected, the multi-locus ML analysis was run using the GTRGAMMA nucleotide substitution model and partition scheme shown in Supplementary Table S3 as estimated with PartitionFinder v. 1.1.1 [66], considering a model with linked branch lengths. Moreover, 1000 rapid bootstrap pseudoreplicates were implemented to evaluate nodal support.

The BI analysis was performed in MrBayes v. 3.2.6 [67]. Optimal substitution models and partition schemes were estimated with PartitionFinder v. 1.1.1 (Supplementary Table S3) [66], considering a model with linked branch lengths and the Bayesian information criterion. The analysis was then conducted with two parallel, simultaneous four-chain runs executed over 5 × 10^7^ generations starting with a random tree and sampling after every 500th step. The first 25% of the data were discarded as burn-in, and the 50% majority-rule consensus tree and corresponding posterior probabilities (PP) were calculated from the remaining trees. The average standard deviation of split frequency values below 0.005 and potential scale reduction factor values approaching 1.00 were considered as indicators of chain convergence.

For visualisation, only BI trees were used. Tree nodes showing bootstrap support (BS) values equal to or higher than 70% and PP equal to or higher than 0.95 were considered as significantly supported. Phylogenetic trees were visualised in FigTree v. 1.4 [68], and Inkscape v. 1.4 was used for the artwork [69].

Single-locus trees were also built with RAxML and MrBayes software following the methodology described above for the multi-locus tree, and the nucleotide substitution models and partition schemes shown in Supplementary Table S3. MEGA v. 11 [70] was employed to calculate the percentage of parsimony-informative sites for each locus.

The Genealogical Concordance Phylogenetic Species Recognition (GCPSR) method [71] was employed to delimit phylogenetic species using three independently inherited loci (ITS–LSU, *tef1-α*, and *rpb2*) using the procedure described by Biketova et al., 2025 [72].

### 2.5. Chemical Analysis

In order to investigate the arsenic accumulation abilities of *Cyanoboletus* species, we used small fragments of basidiomes (80–350 mg) taken from fungarium specimens. These were carefully cleaned from adhered substrate debris using dissection needles, roughly cracked, and weighed in 60 mL perfluoroalkoxy alkane beakers (Savillex, Eden Prairie, MN, USA). Then, 6 mL of 14 mol L^−1^ HNO_3_ was added, the beakers were closed, moved onto a hotplate, and the samples were digested for 16 h at 190 °C. Subsequently, the digests were evaporated to a drop, transferred into volumetric flasks and filled to 25 mL with 2% HNO_3_. Sample digestion was carried out in a clean laboratory housed at the Institute of Geology of the Czech Academy of Sciences (IG CAS) using a HEPA-filtered air environment and class-100 laminar flow hoods. Deionised water with a resistivity of 18.2 MΩ·cm (Milli-Q Element, Merck KGaA, Darmstadt, Germany) was used throughout the sample preparation. The HNO_3_ (65%, J.T. Baker, Phillipsburg, NJ, USA) acid was in-house double Teflon-distilled by a distillation apparatus (DST-1000 and DST-4000, Savillex, USA) before use for sample processing. Arsenic concentrations in the resulting solutions were analysed shortly after dilution (10×) in 2% HNO_3_ by High Resolution Inductively Coupled Plasma Mass Spectrometry (HR-IC-PMS) using the instrument Element 2 (Thermo Scientific, Waltham, MA, USA) housed at IG CAS. Standard analytical conditions of the instrument were utilised to analyse the solutions. Arsenic was quantified via external calibration using blank and monoelement As solutions (EPOND, Effretikon, Switzerland). Indium solution with a concentration of 1 µg In L^−1^ was added via a T-piece in the sample introduction system as the internal standard. Standard reference material SRM 1566b, Oyster Tissue (NIST, Gaithersburg, MD, USA), was processed for quality control of the procedure. Arsenic mass fractions in fungal biomass reported in this paper are expressed on a dry matter basis.

## 3. Results

### 3.1. Molecular Sequence Datasets and Phylogenetic Analyses

Forty-two ITS and six LSU sequences were newly generated in the present study for *C. mediterraneensis*, *C. poikilochromus, C. pulverulentus*, and *C. sinopulverulentus* collections (Supplementary Table S2). The ITS sequence for GS11161 consisted of 114 bp and, therefore, was excluded from sequences submitted to GenBank. Characteristics for the five assembled sequence datasets used for phylogenetic inference are shown in Table 2. The most variable marker was ITS, although the number of ITS sequences was more than double that of the other three markers. The aligned multi-locus matrix is provided in Supplementary File S2.

**Figure 1:**
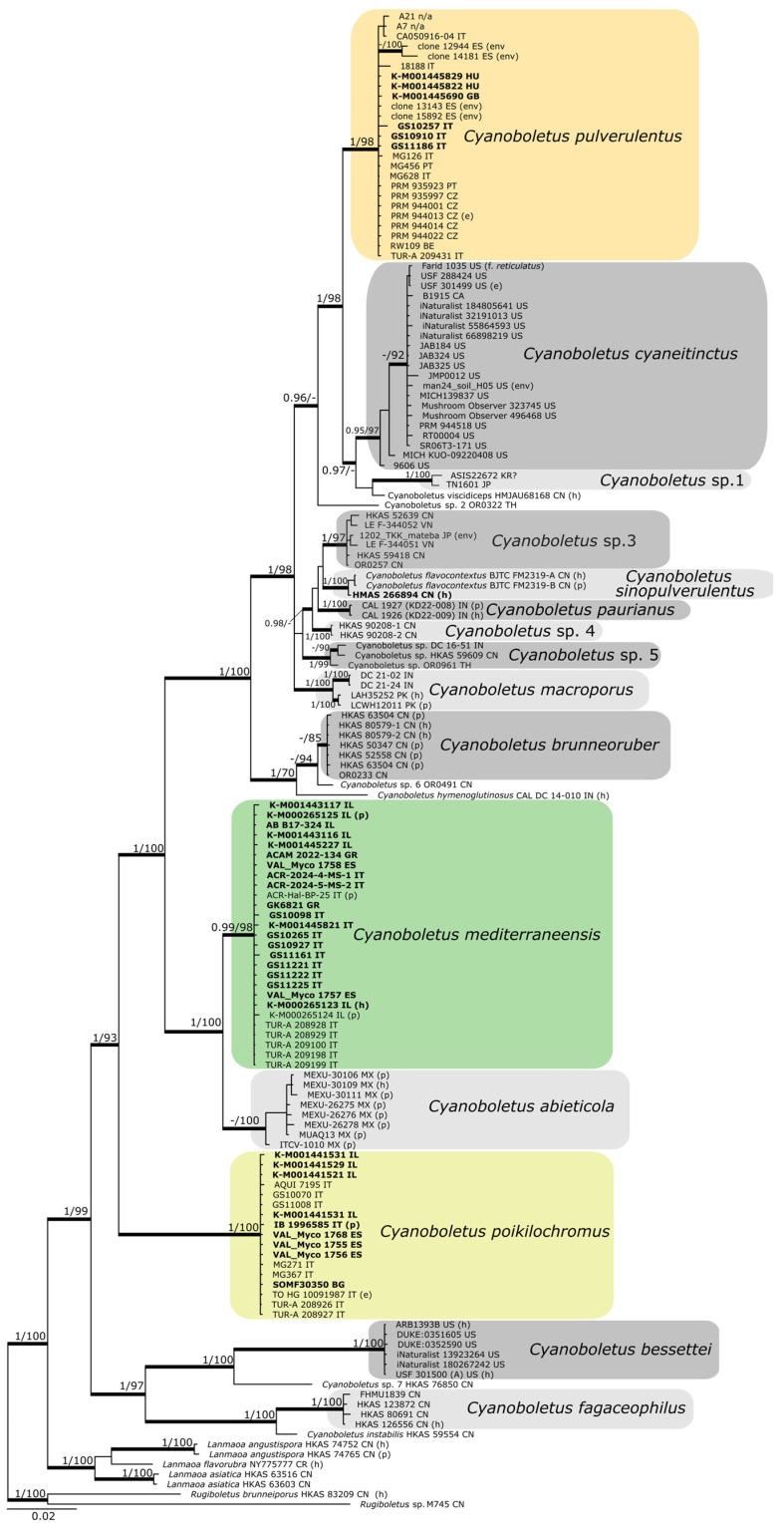
The BI phylogenetic tree of *Cyanoboletus* generated from a multilocus (ITS + LSU + *tef1*-*a* + *rpb2*) dataset. The BS values of ≥70% and the PP values of ≥0.95 are indicated at the nodes. Thickened branches indicate high statistical support (either BS ≥ 70% or PP ≥ 0.95). Species names of collections follow current identification, except original names of the type specimens of *C. flavocontextus*. *Cyanoboletus* species occurring in Europe and the Levant are indicated by coloured fields. Collections with newly generated sequences are indicated in bold. Two-letter country codes (ISO 3166-1[73] alpha-2) denote the origin of the specimens. At the end of the annotations of some collections, abbreviations are indicated in brackets: e—epitype, h—holotype, p—paratype, and env—environmental sample.

The multi-locus phylogeny produced with the ML had an lnL value of −13,425.118793, whereas the BI analysis reached an average standard deviation of split frequencies of 0.005 after 22.4 × 10^6^ generations. Average effective sample sizes (ESSs) were well above 200 in the BI analysis. Because the obtained phylogenies showed no supported conflicts, the topology inferred under a BI framework is presented in Figure 1.

*Cyanoboletus* is confirmed as a strongly supported generic clade (PP = 1.00, BS = 99%) encompassing 21 monophyletic species-level clades based on multi-locus analysis, 14 of which represent known species and seven undescribed taxa. However, according to the GCPSR method based on comparison of phylogenies of three independently inherited loci (ITS–LSU, *tef1-α*, and *rpb2*), we confirm 15 phylogenetic species and five putative phylogenetic species. These numbers are lower than the numbers of clades in the multi-locus phylogeny due to the fact that seven species are missing some of the analysed marker loci.

The majority of previously described *Cyanoboletus* species, including our focal species *C. mediterraneensis*, *C. poikilochromus* and *C. pulverulentus*, formed highly supported clades (PP ≥ 0.99, BS ≥ 98%). Only *C. flavocontextus* is clustered in the same species-level clade as *C. sinopulverulentus*, supporting its conspecificity (PP = 1.00, BS = 100%). The reconstructed phylogeny provided high support for the close relationship between the European *C. pulverulentus* and the North American *C. cyaneitinctus* (common subclade: PP = 1.00, BS = 98%), as well as the mainly Mediterranean *C. mediterraneensis* and the Mexican *C. abieticola* (common subclade: PP = 1.00, BS = 100%). A number of collections formed well-supported clades but without clear affinities to any already described *Cyanoboletus* species. In such cases, we have named them as *Cyanoboletus* sp., followed by a number (1 to 7).

**Figure 2:**
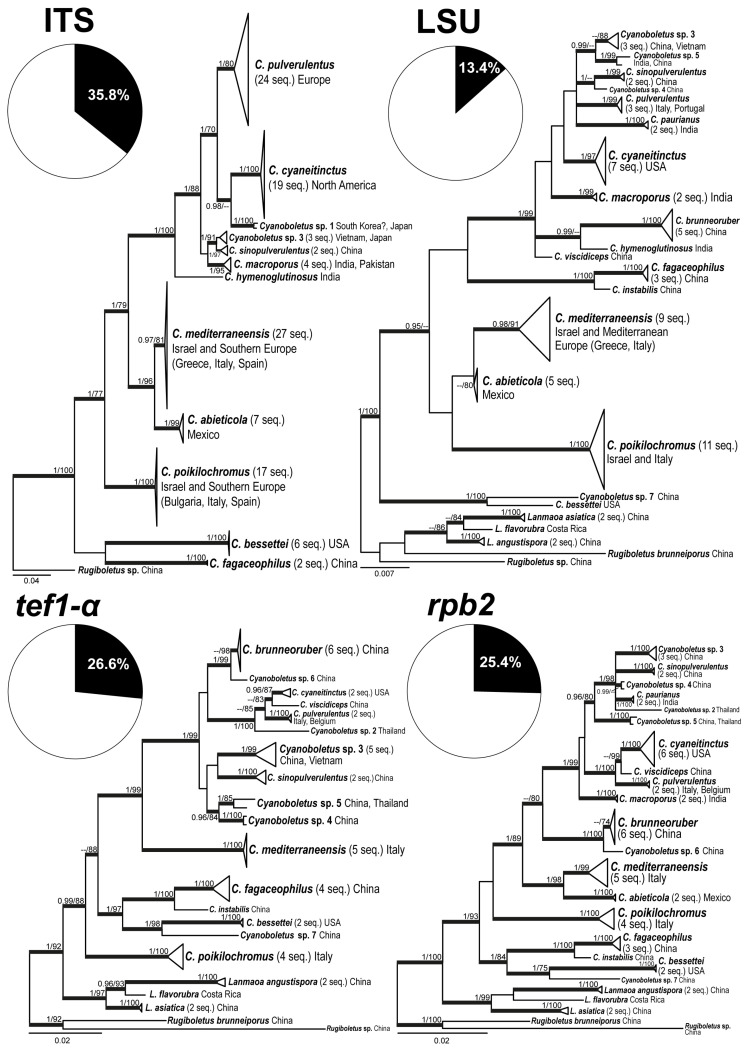
The Phylogenetic trees of *Cyanoboletus* from separate BI analyses of each locus: ITS, LSU, *tef1-a*, and *rpb2*. BS values of ≥ 70% and PP values of ≥ 0.95 are indicated at the nodes. Thickened branches indicate high statistical support (either BS of ≥70% or PP of ≥0.95). The pie charts underneath each label are the percentage of the total characters in the alignment that are parsimony informative.

Single-locus BI phylogenies are shown in Figure 2. The base topology in all four cases matches the one obtained with the multi-locus phylogeny. PP and BS for the inner branches and sister-level relationships were generally high in phylogenies built with ITS, *tef1*-α, and *rpb2* data. As in the multi-locus phylogeny, the study species *C. mediterraneensis*, *C. poikilochromus*, and *C. pulverulentus* formed highly supported clades (PP ≥ 0.97, BS ≥ 80%) in all single-locus phylogenies, and *C. mediterraneensis* was shown to be closely related to *C. abieticola*, and *C. pulverulentus* to *C. cyaneitinctus* and *C. viscidiceps*, whereas the close affinities of *C. poikilochromus* to other taxa remained elusive.

### 3.2. Taxonomy

***Cyanoboletus*** Gelardi, Vizzini & Simonini, in Vizzini, Index Fungorum 176: 1, 2014, emend. Biketova.

MycoBank MB 550672

=*Cupreoboletus* Simonini, Gelardi & Vizzini, in Gelardi, Simonini, Ercole, Davoli & Vizzini, Mycologia 107(6): 1257 (2015); generic type: *Boletus poikilochromus* Pöder, Cetto & Zuccher.

*Diagnosis*: Basidiomes pileate-stipitate with tubular-poroid hymenophore, epigeous, small to medium, and rarely large, evelate; pileus tomentose to glabrous, dry to slightly tacky; hymenophore adnate to adnexed, sinuate or (sub)decurrent, yellow to olive green or rarely orange, yellowish brown, brownish red to reddish brown; stipe surface smooth to pruinose, sometimes with longitudinal striations in the upper half, rarely reticulate; context whitish to yellow, sometimes reddish tinged at the stipe base; tissues instantly discolouring dark indigo blue to blue–black when handled or injured and sometimes fading to copper tints, or showing changes that are much less intense, ranging from light blue or greenish blue to almost unchanging in the stipe; taste mild or slightly acidic; smell inconspicuous, fruity or sometimes peculiar and persistent; spore print olive brown; basidiospores smooth, ellipsoidal to ellipsoidal-fusoid, ellipsoidal-subamygdaliform or narrowly amygdaliform; cystidia cylindrical-fusoid to ventricose-fusoid or lageniform, rarely with abundant gloeocystidia; pileipellis a trichoderm (including intricate trichoderm), ixotrichoderm or ixocutis; hymenophoral trama bilateral divergent of the *Boletus*-type; lateral stipe stratum of the boletoid type; stipe base context inamyloid, rarely weakly amyloid, or occasionally weakly dextrinoid; clamp connections absent; ontogenetic development gymnocarpic.

Generic type: *Boletus pulverulentus* Opat. 1836.

***Cyanoboletus pulverulentus*** (Opat.) Gelardi, Vizzini & Simonini, in Vizzini, Index Fungorum 176: 1 (2014)

**Figure 3:**
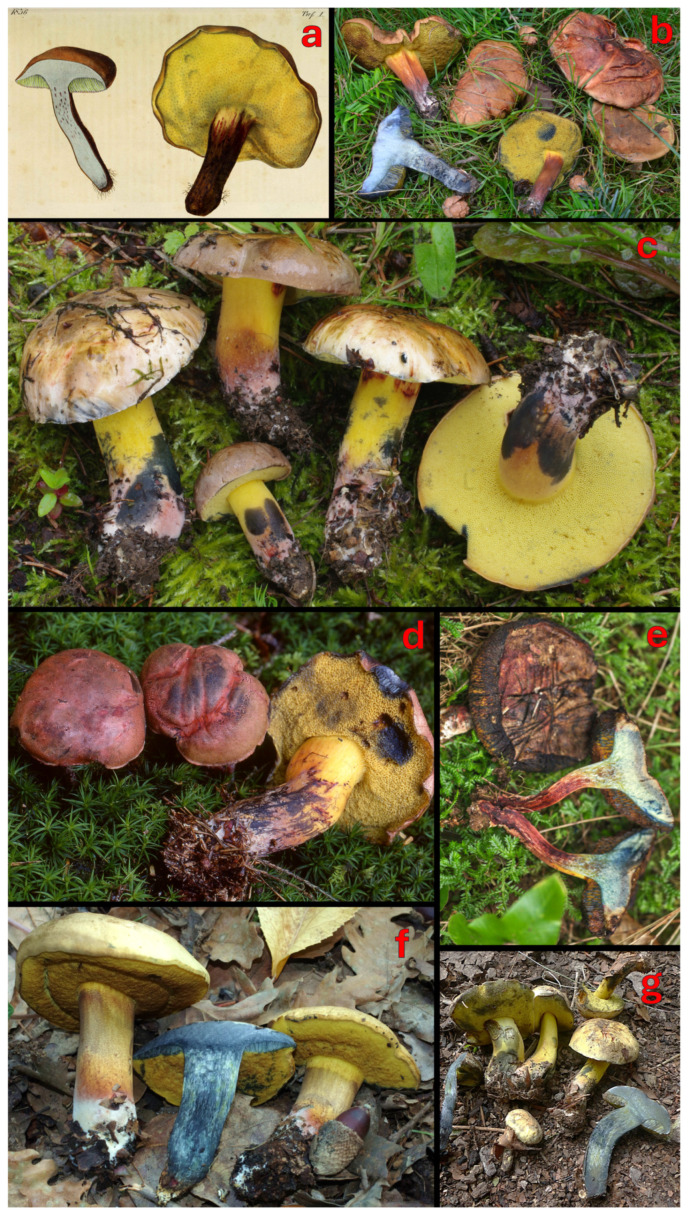
The macromorphology of *C. pulverulentus*: (**a**) an original holotype illustration, (**b**) MG1050, (**c**) an epitype collection PRM 944013, (**d**) GS1551, (**e**) PO-F2601, (**f**) GS10910, and (**g**) K-M001445829 (AB B18-391). Photos and pictures: (**a**) W. Opatowski [74], (**b**) M. Gelardi, (**c**) J. Borovička, (**d**) C. Lavorato, (**e**) V. Fachada, (**f**) G. Simonini, and (**g**) B. Bálint.

**Figure 4:**
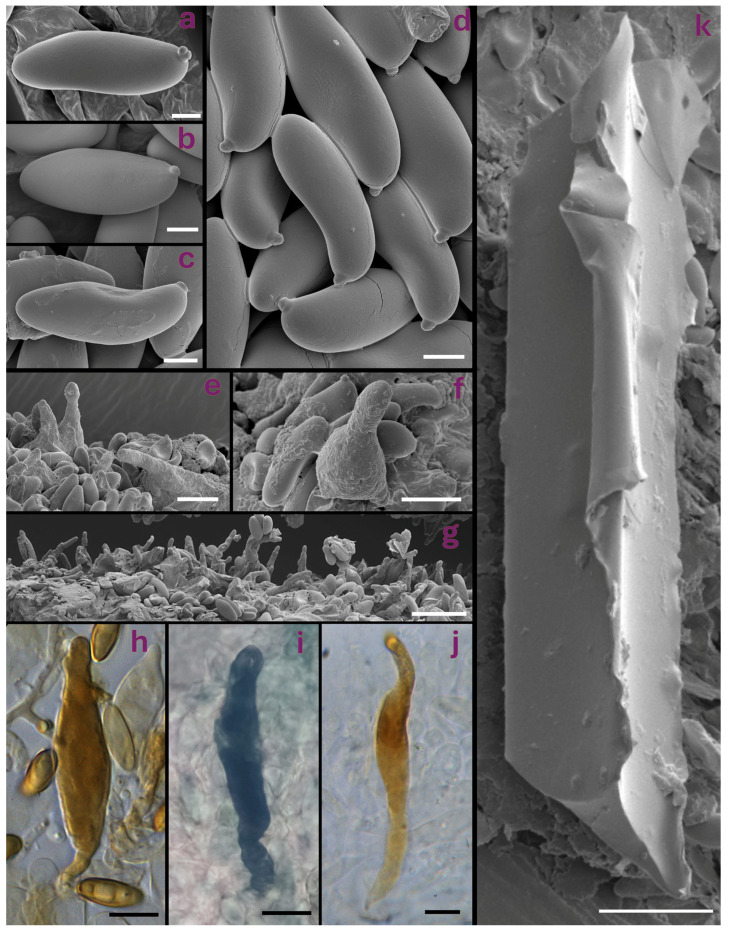
The microscopic features of target *Cyanoboletus* species: (**a**) typical long, narrow-subamygdaliform basidiospore of *C. mediterraneensis* under SEM (PO-F2442); (**b**) typical short, blunt-ellipsoidal basidiospore of *C. poikilochromus* under SEM (MG1004); (**c**) typical narrow-amygdaliform basidiospore of *C. pulverulentus* under SEM with significant suprahilar depression, lateralised apiculus, and with relatively acute apex (PO-F2601); (**d**) basidiospores of *C. pulverulentus* under SEM (PO-F2601); (**e**,**f**) hymenophore of *C. pulverulentus* displaying fusiform-lageniform cystidia under SEM (PO-F2601); (**g**) typical hymenophore of *C. poikilochromus* with abundant narrow-cylindrical gloeocystidia (MG1004); (**h**) gloeocystidium of *C. pulverulentus* with KOH (PO-F2601); (**i**) gloeocystidium of *C. poikilochromus* with brilliant cresyl blue (IB 19960585, paratype); (**j**) gloeocystidium of *C. poikilochromus* with KOH (GS63); (**k**) crystal structure from the hymenophore of *C. poikilochromus* under SEM (MG1004). Bars: 2 μm (**a**–**d**), 10 μm (**e**,**f**,**h**–**j**), and 20 μm (**g**,**k**). Micrographs: (**a**–**h**,**k**) V. Fachada, (**i**) A. Yu. Biketova, and (**j**) G. Simonini.

**Figure 5:**
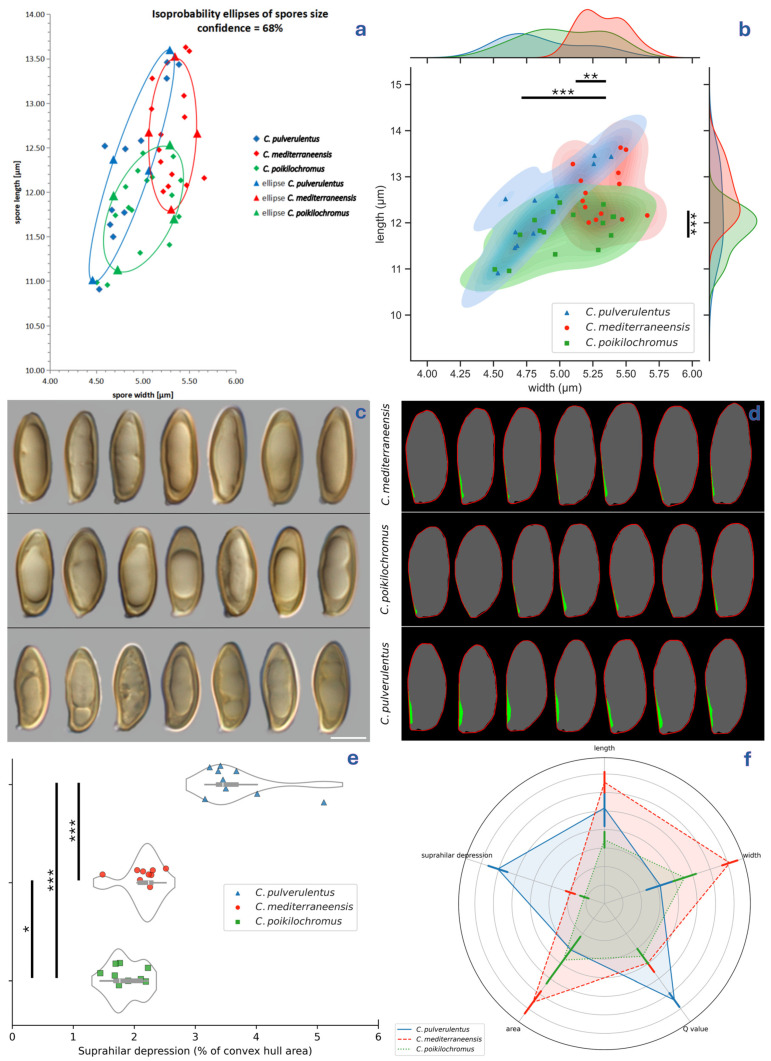
The basidiospore morphometry of target species of *Cyanoboletus*: (**a**) isoprobability ellipses; (**b**) kernel density estimate, where each mark represents a measured collection, ** *p* = 0.01, and *** *p* = 0.001 using *t*-tests; (**c**) the representative basidiospores of each species in the side view; (**d**) segmentation of (**a**) (grey silhouette), generating a respective convex hull (red line) and ultimately their difference, i.e., concave hull area equating to the suprahilar depression (green); (**e**) a violin plot depicting suprahilar depression represented as a percentage of the convex hull area, where each mark represents a measured collection, * *p* = 0.05, and *** *p* = 0.001 using Mann–Whitney tests; and (**f**) a spider chart comparing the main basidiospore morphology traits, where the bars represent the standard errors. Micrographs: (**c**) V. Fachada.

Figure 3, Figure 4c–f,h and Figure 5.

MycoBank MB 550673

≡*Boletus pulverulentus* Opat., Wiegm. Arch. Naturgesch. 2: 27 (1836) (Basionym).

≡*Xerocomus pulverulentus* (Opat.) J. E. Gilbert, Livres Mycol. 3: 116 (1931).

≡*Tubiporus pulverulentus* (Opat.) Imai, Trans. Mycol. Soc. Japan 8(3): 113 (1968).

=*Uloporus mougeotii* Quél., Enchir. Fung.: 162 (1886).

=*Boletus sistotrema* var. *mougeotii* (Quél.) Costantin & L.M. Dufour, Nouv. Fl. Champ. Edn 1: 152 (1891).

=*Uloporus sistotrema* var. *mougeotii* (Quél.) Quél., Fl. Mycol.: 411 (1888).

=*Gyrodon mougeotii* (Quél.) Sacc., Syll. Fung. 9: 160 (1891).

=*Boletus sistotrema* var. *mougeotii* (Quél.) Bataille, Bull. Soc. Hist. Nat. Doubs 15: 43 (1908), nom. illegit.

=*Boletus mougeotii* (Quél.) Bigeard & H. Guill., Fl. Ch. Sup. France: 385 (1909).

=*Boletus hortensis* Smotl., Sber. K. böhm. Ges. Wiss. [1911]: 40 (1912).

=*Tubiporus nigricans* E. A. Herrm., Pilz- und Kräuterfr. 4 (6/7): 124 (1920).

=*Boletus rickenii* Gramberg, Pilz- und Kräuterfr. 4: 226 (1921).

–*Boletus pulverulentus* var. *sublateritius* Guinb., Lannoy & Estadès in Lannoy & Estadès, Docums Mycol. Mém. Hors Sér. 6: 91 (2001), nom. inval., Art. 39.1 (Madrid).

?**–***Boletus hortensis* f. *citrinus* Smotl., Časopis Čs. Houbařů 29(1–3): 31 (1952), nom. inval., Art. 39.1 (Madrid).

?**–***Boletus hortensis* f. *eurothensis* Smotl., Časopis Čs. Houbařů 29(1–3): 31 (1952), nom. inval., Art. 39.1 (Madrid).

?–*Boletus hortensis* f. *lilacinus* Smotl., Časopis Čs. Houbařů 29(1–3): 31 (1952), nom. inval., Art. 39.1 (Madrid).

*Misapplied names:*

–*Boletus radicans* Pers. *sensu* Fr., 1874, Hymenomycetes Europaei: 503.

–*Boletus radicans* Pers. *sensu* Rea, 1922, s. auct.; fide Checklist of *Basidiomycota* of Great Britain and Ireland, 2005.

*Holotype*: Germany, Berlin–Brandenburg: Berlin, the Royal Botanical Garden of Berlin, tab. I, figures 1 and 2 [74].

*Epitype designated here* (MTB10029081): Czechia, Central Bohemia: Rakovník, Jesenice, 50°05′23.1″ N, 13°29′11.0″ E, in mixed forest plantation under *Quercus robur* and *Corylus avellana*, 17.06.2016, leg. & det J. Borovička, PRM 944013, GenBank: ITS—LT714707.

*Edibility*: Considered edible after prolonged cooking [75]; however, it is not recommended for consumption due to high arsenic content in the basidiomes ([40] and this study).

*Ecology and phenology*: Growing solitary or in small groups, on acid to neutral soils during the summer and autumn ((May) June–November)) ([76] and this study). Associated with *Castanea sativa*, *Carpinus betulus*, *Corylus avellana*, *Fagus sylvatica*, *Quercus* (*Q. cerris*, *Q. ilex*, *Q. pubescens*, *Q. robur*), *Tilia cordata*, *Pinus* (*P. nigra*, *P. pinea*, *P. sylvestris*), *Cedrus libani*, and *Tsuga* spp., based exclusively on genetically proven collections ([8,40,59] and this study). *Quercus coccifera*, *Q. pyrenaica*, *Q. suber*, *Alnus*, *Betula*, *Ostrya*, *Abies*, and *Picea abies* are also hosts of reported non-genetically verified collections ([8,77,78,79,80,81] and this study).

*Known distribution*: EUROPE: Andorra [14]; Austria [12,14]; Belgium ([12,14], GP—[82]); Bulgaria ([83,84,85] and this study); Czechia ([13,86,87]; GP—[40] and this study); Croatia [13,88]; Denmark [26,89]; France (incl. Corsica) [13,14,90,91,92,93]; Germany [13,74]; Greece [80,81,94,95,96,97,98,99]; Hungary ([100]; GP—this study); Ireland [101,102]; Italy (incl. Sicily and Sardinia) ([78,103,104,105,106,107]; GP—[8,108,109] and this study); Kosovo* [110]; Lithuania [14,111]; Luxemburg [14]; Malta [112]; Moldova [14]; Netherlands [113,114]; North Macedonia [115,116]; Norway [26,89]; Poland [117,118,119]; Portugal [79]; Romania [120,121,122]; Russia (European part) [123]; Serbia [124,125]; Slovakia [77]; Slovenia [126]; Spain (inc. the Balearic Islands) ([13,78,79,127,128,129]; GP—[130] and this study); Sweden [26,89]; Switzerland ([13,131] and this study); Ukraine [76,132,133]; United Kingdom ([101,102,134,135,136,137]; GP—Janke unpubl. [59] and this study). MACARONESIA: Portugal (Azores, Madeira) ([138,139]; GP—[8,40] and this study); Spain (Canary Islands) [140,141,142,143,144]. WEST ASIA: Iran [145]; Turkey (Balıkesir and Karaman Provinces) [146]. This species is also recorded in AUSTRALIA [14,44]; NORTH AFRICA (Morocco, Tunisia) [12,147,148,149]; NORTH and EAST ASIA (Japan, the Russian Far East, South Korea, Taiwan*) [14,27,28,123,150,151,152,153]; SOUTH ASIA (Pakistan) [154]; NORTH and CENTRAL AMERICA except the Western Group of the Azores (Canada, USA, Mexico, Guatemala) [14,155,156,157,158,159]; and SOUTH AMERICA (Colombia) [14,160,161]. It should be noted that extra-European, extra-Macaronesian, and extra-West Asian records may represent different *Cyanoboletus* species (or even other genera), and their identity should be verified by DNA sequencing.

*Notes*: *Cyanoboletus pulverulentus* was originally described by Opatowski from Germany, based on collections growing in the Berlin Botanical Garden and Botanical Museum (former Royal Botanical Garden of Berlin) [74]. The specific epithet “*pulverulentus*” means “covered with powder” and refers to the somewhat dry powdery surface of the young pileus and the stipe base. The holotype is an iconotype illustrating two basidiomes, where one is longitudinally cut, showing blueing flesh, and the other is in anterolateral view (Figure 3a). Due to the absence of any physical fungal type specimen and because this historical illustration is demonstrably ambiguous, lacking essential microscopic features and DNA data is required for precise modern species identification and phylogenetic placement (Art. 9.9 [162]), here we designate an epitype from a similar habitat in Central Bohemia (Czechia) that matches the original description (Figure 3c).

**Figure 6:**
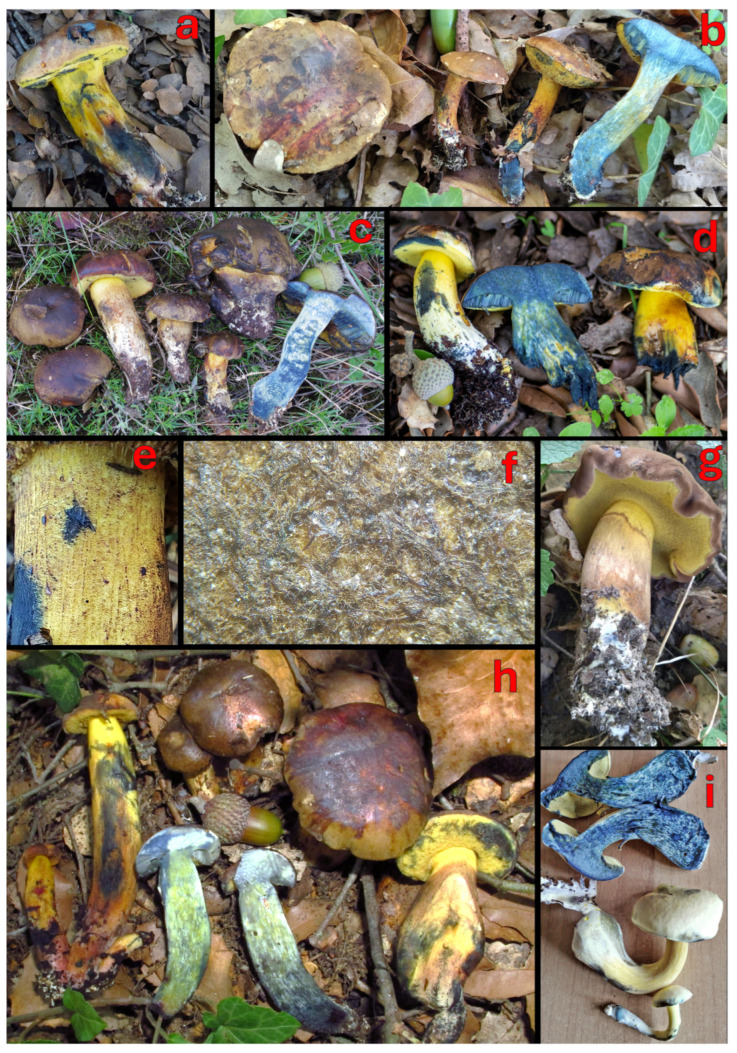
The macromorphology of *C. mediterraneensis*: (**a**) holotype collection K-M000265123 (AB B12-077); (**b**) SOMF 30989; (**c**) GS10265; (**d**) VAL_Myco 1757 (IGB1682); (**e**) pseudoreticulate pattern on the surface of one of SOMF 30989 basidiomes; (**f**) GS11225, pileus surface detail; (**g**) GS11190; (**h**) GK6821; (**i**) holotype of *C. mediterraneensis* f. *pallidus*, MCVE 31989 (PAn1213_13112025). Photos: (**a**) Z. Sahfranov, (**b**,**e**) B. Assyov, (**c**) A. Errico, (**d**) I. Garrido-Benavent, (**f**) M. C. Morosini, (**g**) G. Simonini, (**h**) G. Konstantinidis, and (**i**) P. Angeli.

*Cyanoboletus pulverulentus* is macroscopically characterised by small to medium-sized basidiomes, growing solitary or in groups. The pileus is rather variable in colouration: yellow, buff brown, olivaceus brown to raspberry red, with a silky to slightly viscous surface and an acute pileus margin; the stipe is yellow, sometimes with brown or reddish colours in the lower part, rarely with a reticulum in the upper part [2]; tubes are lemon yellow; the context is yellow, sometimes reddish in the stipe of aged basidiomes, which turns intense blue with a turquoise tint (Figure 3). Microscopically, this species is characterised by narrow-amygdaliform, subfusiform-subamygdaliform, or amygdaliform basidiospores, often with a strongly lateralised apiculus (hilar appendix) and pronounced suprahilar depression, (10.91) 12.29 ± 0.9 (13.46) × (4.53) 4.87 ± 0.32 (5.39) µm, with the largest Q value of the European species of *Cyanoboletus*—(2.41) 2.53 ± 0.18 (2.73) (Figure 4c,d, Figure 5 and Figure 6). Thriving in acidic to neutral soils across temperate European and Macaronesian forests, mycorrhizal with broadleaves and conifers. It also differs from other European *Cyanoboletus* species and *C. cyaneitinctus* by arsenic hyperaccumulation ([40] and this study).

It is difficult to separate *C. pulverulentus* from *C. mediterraneensis* in the field, as both species exhibit variable macromorphological characters, although the latter tends to be confined to thermophilic Mediterranean habitats and often produces a darker pileus colour. Likewise, microscopically, there is a significant overlap in basidiospore length and width, although basidiospores are smaller on average in *C. pulverulentus*. Nonetheless, both species may be distinguished by basidiospore shape in the side view, as *C. pulverulentus* produces a large proportion of basidiospores with a pronounced suprahilar depression, emphasised by the crooked proximal end with a strongly lateralised apiculus. However, basidiospore morphology is highly variable within each basidiome; therefore, much like the apical truncation in *Xerocomellus*, such micromorphological features can be easily overlooked, and it is important to search for basidiospores showing key features before considering them as being absent [163].

Extra-European, extra-Macaronesian, and extra-West Asian records of *C. pulverulentus* likely belong to different taxa. Records from the USA and Canada represent *C. cyaneitinctus*, which is the most common and most similar lookalike of *C. pulverulentus* among *Cyanoboletus* species in North America [17]. All illustrated records from Mexico on GBIF likely represent a different xerocomoid genus with a reticulated stipe [14]. Reports of this species (as *Boletus pulverulentus*) from the Kamchatka and Sakhalin regions of Russia, judging from descriptions and illustrations in Bulakh [27,28,150], likely represent another species or perhaps even a distinct xerocomoid genus with felty reddish-brown pileus (sometimes with fine cracks), larger, more angular pores, and fibrillose vinaceous stipe surface. The records of a soil sample TUE000376 (sequences UDB04486503, UDB04486504, UDB04486505) of this species from China and specimens from Japan TN1601 (sequence LC832002) and South Korea ASIS22672 (sequences KP004920 and KF668326) belong to the undescribed *Cyanoboletus* sp. 1 (Figure 1 and Figure 2) [14,164]. The collection from Australia (https://www.gbif.org/occurrence/4507683076 (accessed on 17 February 2026)) also represents another species (or even genus) with a shorter hymenophore and more minute and rounded pores than in the genuine *C. pulverulentus* [14]. Some European, Macaronesian, and West Asian records can represent *C. mediterraneensis* or other bolete species, such as the case with a record from Kosovo* by Karadelev et al. [165], which likely represents *Neoboletus xanthopus* (Klofac & A. Urb.) Klofac & A. Urb. based on the photo.

***Cyanoboletus mediterraneensis*** **f.** ***mediterraneensis*** Biketova, Rinaldi & Simonini, Index Fungorum 516: 1 (2022)

Figure 4a, Figure 5 and Figure 6.

MycoBank MB 552946

*Misapplied names:*

–*Boletus pulverulentus* Opat. *sensu* Biketova et al., Plant Biosyst. 150(5): 883 (2016).

*Holotype* (MBT10006276): Israel, Upper Galilee (Northern District): Goren Park, solitary basidiome under *Quercus calliprinos*, 01.12.2012, leg. Z. Shafranov & A. Yu. Biketova, det. A. Yu. Biketova, K-M000265123 (ex herb. HAI B12-077), GenBank: ITS—PZ244171, LSU—NG_228932.

*Basidiomes* small to medium-sized, growing solitary, in groups, or caespitose. *Ontogenetic development* gymnocarpic. *Pileus* 5.0–10.0 cm diam., convex, tomentose, matt, and dry, ranging from yellowish brown, buff brown to hazel, snuff brown, or dark brown, and becoming dark blue–black when bruised; pileus margin slightly incurved to involute, obtuse, and sometimes wavy. *Stipe* 7.0–9.0 × 1.0–2.8 cm, cylindrical, tapered, sometimes rooting, lemon yellow, often brownish to dark brick red at the base, without a reticulum or rarely with a pseudoreticulate pattern (Figure 6e); stipe surface becoming intensely blue–black after handling; basal mycelium white. *Context* rather dense, initially lemon yellow or greenish yellow, becoming intensely dark blue or cobalt blue–black when exposed to air and then gradually fading to greenish; context under tubes lemon yellow, becoming blue–green to dark blue when exposed to air. *Tubes* up to 12 mm long, adnate and subdecurrent, initially lemon yellow, then yellow olivaceous, turning blue–green or blue–black when bruised. *Pores* medium-sized, angular or irregular, lemon yellow to lemon chrome, becoming dark blue when injured. *Spore print* dark olive to brown olive. *Smell* slightly acidulous. *Taste* mild, slightly acidulous.

*Basidiospores* [703/14/17] (12.01) 12.67 ± 0.74 (13.63) × (5.10) 5.33 ± 0.32 (5.66) μm, Q = (2.15) 2.38 ± 0.15 (2.61), A = (50.7) 57.8 ± 5.6 (66.4) μm^2^, apV = (171.9) 189.7 ± 29.1 (216.2) μm^3^, long narrowly subamygdaliform, narrowly amygdaliform, and sometimes ellipsoid-fusiform to fusiform-subamygdaliform, smooth, thick-walled, guttulate, and light yellow–brown. *Basidia* 27–35 × 7–10 μm, 4-spored, clavate, hyaline, guttulate, without basal clamp. *Hymenial cystidia* 38–71 × 10–14 μm, fusiform, ventricose or lageniform, ending with papilla, some with intracellular yellowish oil content. *Hymenophoral trama* of the *Boletus* type. *Caulocutis* fertile. *Caulocystidia* 25–52 × 7–14 μm, fusiform, claviform or ventricose, some sinuous. *Pileipellis* is a trichoderm or ixotrichoderm of intertwined septate hyphae, consisting of cylindrical, filamentose cells (3.5–) 5–6 (–7.5) μm wide, mostly widely and finely incrusted with yellow–brown pigment.

*Macrochemical spot-test reactions:* 25% NH_4_OH: dark blue context becomes light fulvous and pileus surface becomes dark sienna; 30% KOH: context becomes light fulvous, and pileus surface becomes dark brick; 10% H_2_SO_4_: context becomes clay pink and later yellow, and pileus surface becomes sienna; 10% FeSO_4_: context fades to greenish yellow or lemon yellow; Melzer’s reagent: context in the stipe base is inamyloid (I^−^).

*Edibility*: Edible after prolonged cooking, although with a somewhat acidic aftertaste.

*Ecology and phenology*: Solitary, in small groups or caespitose, growing in thermophilic Mediterranean forests on neutral or calcareous soil and in cistaceous Mediterranean low-maquis on preferably acidic, sandy soil; associated with *Quercus* spp. (*Q. calliprinos*, *Q. cerris*, *Q. coccifera*, *Q. faginea*, *Q. ilex*, *Q. robur*, *Q. rotundifolia*, *Q. suber*), *Carpinus orientalis*, *Halimium halimifolium*, and *Pinus* spp. (*P. halepensis* and *P. pinea*) ([9,10,13,164,166] and this study). It was also collected in mixed forests and woodlands with the presence of *Arbutus unedo*, *Cistus* spp. (*C. albidus* and *C. salvifolius*), and *Populus* ([13] and this study). Rare throughout its distribution range but likely largely overlooked.

*Known distribution*: EUROPE (Southern Europe): Bulgaria (Varna Province) (GP—this study), France (Corsica) [167], Greece (Crete, Epirus) ([168]; GP—this study), Italy (Apulia, Emilia Romagna, Lazio, Ligury, Lombardy, Marche, Sardinia, Sicily) ([13]; GP—[164], [166] (as *Boletus* sp.), Carbone et al. unpubl. [59], and this study); Portugal (Lisboa e Vale do Tejo) (GP—this study), Spain (Andalusia, Valencian Community) ([13]; GP—this study); NORTH AFRICA: Italy (Pantelleria Island) (GP—[164], from soil samples); WEST ASIA (Levant): Israel ([9], as *B. pulverulentus*; GP—[10] and this study).

***Cyanoboletus mediterraneensis*** **f.** ***pallidus*** Angeli, Baldazzi, Gelardi & Biketova, **f. nov.**

Figure 6i.

MycoBank MB 862639

*Holotype* (MBT10031659): Italy, Marche: Rimini, Villa Verrucchio, Via Farneto 25, in a private garden, 43°59′59.3″ N 12°26′39.9″ E, 126 m, three basidiomes growing with *Salix* sp., *Rosa* hybrid, *Jasminum* sp., *Quercus* sp. (30 m away), 13.11.2025, leg. P. Angeli & L. Baldazzi, det. M. Gelardi, MCVE 31989 (collector’s number PAn1213_13112025), GenBank: ITS—PZ244160.

*Etymology*: “*pallidus*” means “pale-coloured”.

*Diagnosis*: Differs from the type form by light-buff to light-yellow pileus, very pale-yellow pores and light-yellow stipe.

*Notes on Cyanoboletus mediterraneensis*: The original specimen belonging to *C. mediterraneensis* was first published by Biketova et al. in 2016 under the misapplied name *Boletus pulverulentus* [9]. This collection (ex herb. HAI B12-077, currently K-M000265123), consisting of a single basidiome, was found in Goren Park in Israel, growing in association with *Q. calliprinos* [9]. Later, this collection was chosen as the holotype of *C. mediterraneensis* [10]. In another study by Leonardi and coauthors, this species was recorded under the name *Boletus* sp., growing in cistaceous Mediterranean maquis on sandy soil in association with *H. halimifolium* in Sardinia, Italy [166]. This collection (ACR-Hal-BP-25) of A. Rinaldi was designated as a paratype of *C. mediterraneensis* [10].

The xanthoid form of *C. mediterraneensis*, lacking brown pigments, is apparently very rare and has been detected only once (a single collection of three basidiomes) in Italy (Marche) (Figure 6i). Therefore, it is difficult to judge how stable this feature is and whether it is genetically determined or possibly caused by external factors. At the same time, the almost totally yellow basidiomes of *C. pulverulentus* have some traces of brown pigment either on the central part of the pileus or the stipe surface, and basidiomes of the same collection can vary in colouration—from completely lemon yellow to slightly brownish (Figure 3f,g). Moreover, their pores have an intense lemon-yellow colour, unlike those of *C. mediterraneensis* f. *pallidus*, which are very pale yellow with a greenish tint or close to cream colour.

Initially, prismatic crystals on the hymenophore surface were detected in collection K-M000265124 under SEM [10]. However, during the investigation of additional collections (K-M001443116, K-M000265125, K-M001445227 and PO-F2442) under SEM, these crystals have not been observed.

The morphological delimitation of *C. pulverulentus* and *C. mediterraneensis* can be defined as critical. As often happens between similar boletes, there is not a single stable discriminatory character, and identification must be the result of careful examination of combined features. Besides that, *C. mediterraneensis* also shares similarities with *Lanmaoa fragrans* (Vittad.) Vizzini, Gelardi & Simonini.

(1) Basidiome appearance:

*C. mediterraneensis* frequently occurs with specimens aggregated at the base (caespitose) and often features medium to medium–large basidiomes. This habit, combined with a long-lasting involute pileus margin and sharing the common habitat, can sometimes lead to confusion with *L. fragrans*. However, this ambiguity is easily resolved by observing the blue oxidation upon cutting, which is quicker and significantly more intense in *C. mediterraneensis* than in *L. fragrans*. Also, the last one has a distinct yellow-chrome colouration in the lower part of the stipe context.

(2) Pileus surface:

The pileus surface, although not showing significant differences in a microscopic analysis, has a tendency to remain felty in *C. mediterraneensis* (e.g., Figure 6f), compared to *C. pulverulentus*, which is more often slightly viscid to the touch. Under very humid climatic conditions, both species show a viscid surface. The colours appear much more variable in *C. pulverulentus*, ranging from yellow to raspberry red, passing through brownish tones, while also exhibiting olive shades. In contrast, the pileus colour of *C. mediterraneensis* f. *mediterraneensis* ranges within a narrow spectrum of brown tones, more or less dark, sometimes with yellowish patches. However, the xanthoid form C. *mediterraneensis* f. *pallidus* has a very light-coloured pileus: light buff to light yellow.

(3) Pileus margin:

The pileus margin tends to be more straight and acute in *C. pulverulentus*, but more involute, obtuse, and wavy (sometimes similar to that in *L. fragrans*) in *C. mediterraneensis* (Figure 6g).

(4) Stipe colour:

To date, innate red tints have been observed on the stipe surface of *C. mediterraneensis* in a single basidiome of collection MG689; these only appear occasionally in specimens in unfavourable conditions. Normally, the stipe surface of *C. mediterraneensis* is yellow, dull yellow, or ochre yellow in the upper part, and slightly browning due to age towards the base. The stipe surface of *C. pulverulentus* can sometimes exhibit evident reddish areas in the lower half to the lower third.

(5) Context discolouration on exposure:

Both species have a quick, strong and intense colour change to blue, but while the context is lemon yellow in *C. pulverulentus*, it appears whitish to pale yellow in *C. mediterraneensis*. The resulting chromatic effect is therefore an intense blue with greenish shades that lean towards dark turquoise in *C. pulverulentus* and a deep ink blue in *C. mediterraneensis*. However, this cannot be used as a key distinguishing feature due to intraspecific colour variations (see Figure 3 and Figure 6), and should be evaluated in combination with other morphological characters.

(6) Basidiospore size:

*Cyanoboletus mediterraneensis* has the largest basidiospores among the European *Cyanoboletus* species. However, the basidiospore size overlaps to some extent among these species (see Figure 5 and Supplementary Table S1). The statistical analysis shows a significant difference in (a) basidiospore width, which is wider in *C. mediterraneensis* (average 5.3 ± 0.3 µm) and narrower in *C. pulverulentus* (average 4.9 ± 0.3 µm), and (b) Q ratio, which is stouter in *C. mediterraneensis* (average 2.38 ± 0.15) and more slender in *C. pulverulentus* (average 2.53 ± 0.18).

(7) Basidiospore shape:

Basidiospores of *C. mediterraneensis* are long, narrowly amygdaliform, ellipsoid-fusiform, and sometimes fusiform-subamygdaliform, with an occasional obvious suprahilar depression, though much less common and pronounced than that of *C. pulverulentus*. Basidiospores of *C. pulverulentus* often show a more acute apex when compared to the other European species, particularly *C. poikilochromus* (Figure 4a–d and Figure 5).

***Cyanoboletus poikilochromus*** (Pöder, Cetto & Zuccher.) M. Carbone, D. Puddu & P. Alvarado, Index Fungorum 534: 1 (2023)

**Figure 7:**
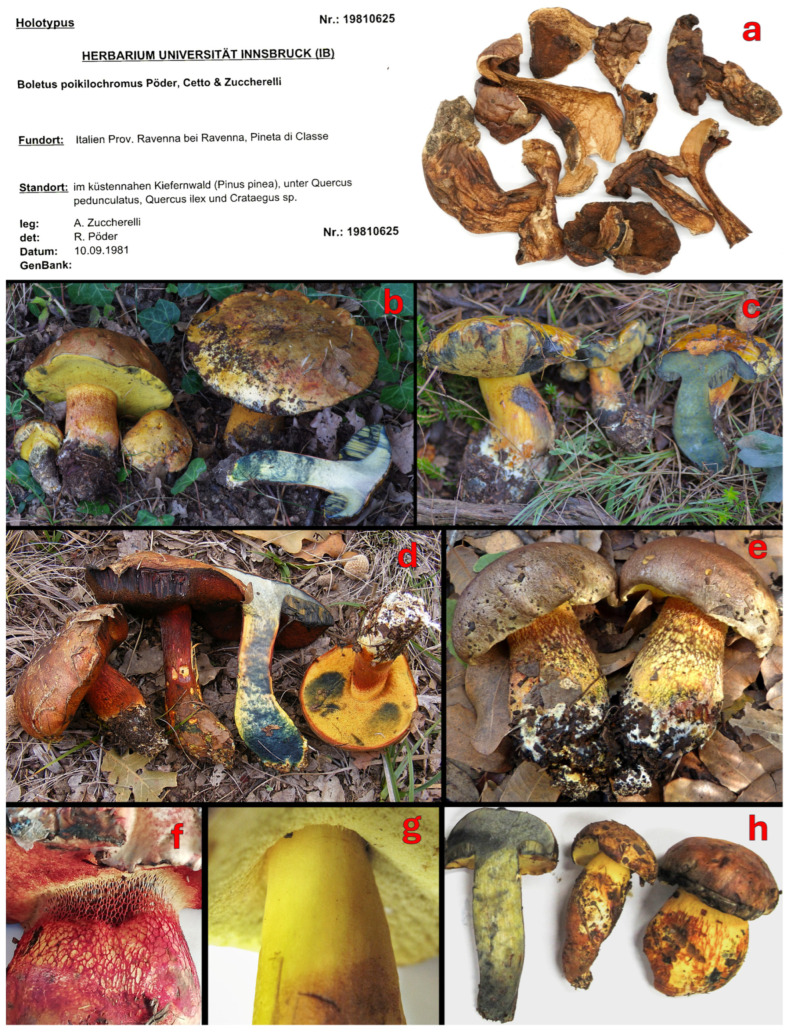
The macromorphology of *C. poikilochromus*: (**a**) holotype collection IB 19810625, (**b**) GS10070, (**c**) VAL_Myco 1756 (IGB1673), (**d**) SOMF 30350, (**e**) K-M001441529 (AB B12-070), (**f**) GS11063, and (**g**,**h**) K-M001441523 (AB B15-262). Photos: (**a**) M. Baldauf, (**b**) G. Simonini, (**c**) I. Garrido-Benavent, (**d**) B. Assyov, (**e**) O. Godorova, (**f**) E. Ponzi, and (**g**,**h**) A. Yu. Biketova.

Figure 4b,g,i–k, Figure 5 and Figure 7.

MycoBank MB 556234.

≡*Boletus poikilochromus* Pöder, Cetto & Zuccher., in Pöder, Mycol. Helv. 2(2): 156 (1987). (Basionym)

≡*Cupreoboletus poikilochromus* (Pöder, Cetto & Zuccher.) Simonini, Gelardi & Vizzini, in Gelardi, Simonini, Ercole, Davoli & Vizzini, Mycologia 107(6): 1257 (2015).

≡*Suillellus poikilochromus* (Pöder, Cetto & Zuccherelli) Blanco-Dios, Index Fungorum 211:1 (2015).

=*Boletus martaluciae* Pacioni, Micol. Veg. Medit. 11(2): 91 (1996).

–*Boletus pulverulentus* f. *reticulatipes* Cetto, I Funghi dal Vero 4: 477 (1983), nom. inval., Art. 39.1 (Madrid).

–*Boletus pulverulentus* f. *reticulatipes* Cetto, Enzyklopädie der Pilze, 1: 535 (1987), nom. inval., Art. 39.1 (Madrid).

*Holotype* (MBT77098): Italy, Emilia Romagna: Ravenna, Pineta di Classe, 44°21′31″ N, 12°16′49″ E, 5 m, with *P. pinea*, *Q. robur*, *Q. ilex*, and *Crataegus* sp., 10.09.1981, leg. A. Zuccherelli, det. A. Zuccherelli & R. Pöder, IB 19810625.

*Epitype* (MBT201639): Italy, Emilia Romagna: Ravenna, Pineta di Classe, with *P. pinea*, *Q. robur*, *Q. ilex*, and *Crataegus* sp., 10.09.1987, leg. A. Zuccherelli, TO HG10091987, GenBank: ITS—KT157047, LSU—KT157056.

*Edibility*: It is considered edible after prolonged cooking (15–20 min) and after pouring out the broth, due to the potential toxic compounds that are either water-soluble or heat-sensitive, as in some other boletes. However, because of its rarity and the intense odour, it is not recommended for consumption [9,75].

*Ecology and phenology*: Solitary or in small groups, ECM in thermophilous broad-leaved and mixed forests in Mediterranean regions, on basic, calcareous and sandy soils, associated with *Quercus* spp. (*Q. alnifolia*, *Q. calliprinos*, *Q. cerrioides*, *Q. coccifera*, *Q. faginea*, *Q. ilex*, *Q. ithaburensis* ssp. *macrolepis*, *Q. pedunculatus*, *Q. pubescens*, *Q. robur*, *Q. rotundifolia*, *Q. suber*), *Pinus* spp. (*P. brutia*, *P. halepensis*, *P. pinaster*, *P. pinea*, *P. sylvestris*), *Ostrya carpinifolia*, often with the presence of *Arbutus unedo*, *Cistus albidus*, and *Crataegus* sp. ([8,9,78,79,169,170,171,172,173,174,175,176] and this study).

*Known distribution*: EUROPE (Southern Europe): Bulgaria (Haskovo Province) (GP—this study); Croatia [177,178]; France (Corsica, Provence-Alpes-Côte d’Azur) ([179] and this study); Greece ([99,168] and this study); Italy (Abruzzo, Apulia, Calabria, Emilia Romagna, Lazio, Marche, Tuscany, Sardinia, Sicily, Veneto) ([2,75,78,107,169,170,180,181,182,183,184,185,186,187,188,189,190,191]; GP—[8] and this study); Portugal (Baixo Alentejo) [79]; Slovenia [126]; Spain (Andalucia, Balearic Islands, Castile-La Mancha, Catalonia, Valencian Community) ([78,79,90,129,171,173,175,176,192,193,194], GP—this study); WEST ASIA: Cyprus [174]; Israel ([9]; GP—this study).

*Notes*: This species was initially described as *Boletus pulverulentus* f. *reticulatipes* (nom. inval.) from Italy by Cetto [195] and was probably confused with *Alessioporus ichnusanus* (Alessio, Galli & Littini) Gelardi, Vizzini & Simonini, to which the same name was initially attributed [196]. *Boletus poikilochromus* was described by Pöder, Cetto and Zuccherelli as a separate species in 1987 [169], based on specimens collected in the Italian region Emilia Romagna. Later, Blanco-Dios placed this species into *Suillellus* Murrill, without any solid justification (likely due to tissues changing to a deep blue when cut and the presence of the reticulum on the stipe) [197]. Then Gelardi et al. [8] introduced a new genus, *Cupreoboletus* Simonini, Gelardi & Vizzini, sister to *Cyanoboletus*, to accommodate this species. Finally, Carbone et al. [11] transferred it to *Cyanoboletus* in 2023; this placement has been confirmed by recent phylogenetic reconstructions of García-Jiménez et al. [24], Zhang et al. [198], and our phylogenetic analyses.

*Cyanoboletus poikilochromus* is macroscopically characterised by medium to medium–large basidiomes; dry or slightly viscid pileus, which is finely tomentose to glabrous, rarely areolate, sometimes cracked, initially buff, dull yellow, yellow ochre, yellowish brown or pale olivaceous brown, becoming ochraceous orange, ochraceous reddish, and dark reddish brown with scattered paler or tawny-red patches; initially involute pileus margin, then progressively expanding and sometimes uplifted, faintly wavy/lobed to regular; lemon-yellow stipe in the upper part, downwards progressively reddish brown to rusty brown, with yellow, cinnamon-brown, reddish-brown, or red reticulum, rarely without it (Figure 7e–h); white basal mycelium; roundish, relatively small pores, which are lemon yellow to slightly rusty orange and rarely red; yellow context; tissues turning intense blue with a turquoise tint then fading copper red to copper brown ([8,9,13] and this study).

*Cyanoboletus poikilochromus* can also be separated from other European and Western Asian *Cyanoboletus* spp. by its short, ellipsoidal basidiospores, (10.96) 11.83 ± 0.69 (12.44) × (4.51) 5.03 ± 0.25 (5.41) μm, Q = (2.16) 2.36 ± 0.16 (2.51), often with a blunt apex and poorly defined suprahilar depression (see Figure 4b and Figure 5). Other remarkable microscopic features of *C. poikilochromus* include: (a) numerous gloeocystidia (so-called “pseudocystidia” or subhymenial cystidia)—sterile honey-yellow cells with oily content, refractile, most often originating from the subhymenium and connected with oleiferous hyphae, characterised by strong absorption of brilliant cresyl blue, dextrinoid reaction with Melzer’s reagent, and that presumably have secretory function (Figure 4g,i,j); (b) presence of glassy, needle-shaped crystals forming on the hymenophore and stipe surface in dry specimens (Figure 4k) [8,9,170]. Moreover, *C. poikilochromus* has a very peculiar, persistent smell that resembles that of propolis, cinnamon, fermenting fruits, or poplar (*Populus nigra*) flower buds and cannot be confused with any other European bolete [8,9,170]. These remarkable features (fading copper-red to copper-brown tissues, peculiar smell, numerous gloeocystidia and crystals) can be connected to some chemical compounds, which are actively produced in this species.

Hyphae of the context in the stipe base of *C. pulverulentus* and *C. mediterraneensis* have an inamyloid reaction with Melzer’s reagent, the same as the majority of studied collections of *C. poikilochromus*, which sometimes have a positive (mostly weak) amyloid reaction [9,169]. Ecologically, *C. poikilochromus* may be found frequently on more alkaline substrates, despite sharing a similar geographic range with the more acidophilic *C. mediterraneensis*.

Therefore, *C. poikilochromus* is very easily recognisable in the field. However, rare misidentifications also happen, e.g., the first mention of this species in Croatia by Božac [199] was attributed to a photo of a bolete of another genus, likely *Suillellus* or *Rubroboletus*.

We studied the holotype IB 19810625 (Figure 7a), paratype IB 19960585, and epitype TO HG10091987 collections of this species in two different laboratories in parallel. Unfortunately, attempts to amplify the ITS and LSU regions of the holotype have failed. However, the sequencing of the paratype collection IB 19960585 resulted in a full-length ITS (PZ244176) and partial LSU (PZ231929) sequences. Therefore, the epitype designation in Gelardi et al. [8] can be considered redundant.

*Key to the Described Mediterranean Species of the Genus* Cyanoboletus

**I. Macromorphological and ecological key:**

1. Stipe with a well-defined reticulum (at least in the large majority of specimens); rounded pores; a peculiar smell that resembles those of fermented fruits; growing preferably on calcareous soil in Mediterranean habitats ………………………………………………………………………………………………………………………………………………………………………….***C. poikilochromus***

1. Stipe devoid of reticulum (or rarely with a fine reticulum restricted to the stipe apex or with a pseudoreticulate pattern); angular or irregularly-arranged pores; with an indistinct mushroomy smell …………………………………………………………………………………………………………………………………………………………………………………………………………….**2**

2. Pileus often slightly viscous or silky, with yellow, buff-brown, olivaceus-brown, or raspberry-red surface; pileus margin straight and acute; stipe sometimes with red tones; context lemon yellow and quickly turns dark blue with greenish shades; occurring throughout Europe, Macaronesia, and West Asia, mainly in temperate habitats with a wide range of *Fagaceae*, *Betulaceae*, *Pinaceae* and *Tilia*……………………………………………………………………………………………………………………………………………………………………………………………***C. pulverulentus***

2. Pileus persistently felty; pileus margin involute, obtuse or wavy; stipe generally lack innate red tones; context whitish or pale yellow, turns deep ink blue usually without evident greenish shades; growing in Mediterranean thermophilic, preferably acidophilic habitats with *Quercus*, *Pinus* or *Cistaceae*……………………………………………………………………………………………..**3**

3. Pileus snuff brown, dark brown, or buff brown; pores and stipe lemon yellow, often brownish to dark brick red at the base……………………………..***C. mediterraneensis*** **f.** ***mediterraneensis***

3. Pileus light buff to pale yellow; pores pale yellow; stipe light yellow without any brown or red shades………………………………………………………………..***C. mediterraneensis*** **f.** ***pallidus***

**II. Microscopy key, to be applied to mature basidiospores in side view only:**

1. Average basidiospore width < 5 µm …………………………………………………………………………………………………………………………………………………………………………………**2**

1. Average basidiospore width > 5 µm …………………………………………………………………………………………………………………………………………………………………………………**4**

2. Average basidiospore length > 12.5 µm …………………………………………………………………………………………………………………………………………………………..***C. pulverulentus***

2. Average basidiospore length < 12.5 µm ……………………………………………………………………………………………………………………………………………………………………………..**3**

3. Basidiospores mostly ellipsoid with poorly defined suprahilar depression and somewhat blunt apex; gloeocystidia abundant…………………………………………………..***C. poikilochromus***

3. Basidiospores often narrowly amygdaliform, with pronounced lateralised apiculus, pronounced suprahilar depression, and with relatively acute apex; gloeocystidia sparse………………………………………………………………………………………………………………………………………………………………………………………………………..***C. pulverulentus***

4. Average basidiospore length < 12.5 µm, mostly ellipsoidal; gloeocystidia abundant …………………………………………………………………………………………………….***C. poikilochromus***

4. Average basidiospore length > 12.0 µm, often narrowly subamygdaliform or amygdaliform with more or less evident suprahilar depression; gloeocystidia sparse……………………………**5**

5. Basidiospores often narrowly amygdaliform with strongly lateralised apiculus and pronounced suprahilar depression (generally > 3% of convex hull area)………………….***C. pulverulentus***

5. Basidiospores narrowly subamygdaliform, with only occasional obvious suprahilar depression (generally < 2.5% of convex hull area)……………………………………….***C. mediterraneensis***

*Extralimital Taxa*

***Cyanoboletus sinopulverulentus*** (Gelardi & Vizzini) Gelardi, Vizzini & Simonini, in Vizzini, Index Fungorum 176: 1 (2014).

MycoBank MB 803339.

*≡Boletus sinopulverulentus* Gelardi & Vizzini, Sydowia 65(1): 49 (2013).

=*Cyanoboletus flavocontextus* L. Fan, N. Mao & T.Y. Zhao, in Mao, Zhao, Zhang, Li, Lv & Fan, Mycosphere 14(1): 2034 (2023).

*Holotype* (MBT174647): China, Shaanxi Province: Qinling Mountains, Heihe National Natural Forest Park, Yingbanliang village, 1432 m, on very moist and drained soil under *Castanea mollissima*, 30.09.2011, leg. M. Gelardi & J.-Z. Sun, HMAS 266894 (isotypes: TO HG2821, MG434), GenBank: ITS—PZ244192.

*Notes*: *Cyanoboletus sinopulverulentus* is characterised by small to medium-sized basidiomes; dark-brown pileus with glabrous to subtomentose surface; dark-brown stipe, either with glabrous yellow to yellow–brown surface in the upper part that gradually goes brown towards the stipe base, or transversely streaked scissurate in the upper half with yellowish ground colour visible in the cracks; whitish or pale-yellow context in pileus to light yellow in stipe and reddish at the stipe base, turning intensely indigo blue; unstuffed small roundish to angular pores, first bright yellow or dull yellow and then turning orange–yellow with age; ellipsoid-fusiform basidiospores, 11.5–13.5 × 4.5–5.7 μm, Q = 2.31 ± 0.14; both 2- and 4-spored basidia are common. This species is growing in association with *C. mollissima* and *Quercus* sp. in temperate montane forests [3,20].

*Boletus sinopulverulentus* was described from Shaanxi province of China by Gelardi et al. in 2013 [3]. There is a lot of confusion in the recognition and identification of this species. Two later records of this species reported as *C. sinopulverulentus* from China (HKAS 59609: KF112366, KF112193, KF112700) and India (DC 16-51: MH684757) [4,15,40] represent another species—*Cyanoboletus* sp. 5, based on our phylogenetic reconstructions (Figure 1 and Figure 2).

In this study, we generated a high-quality ITS sequence PZ244192 of the holotype of this species (HMAS 266894); the previously generated sequence KC579402 [3,59] had a few poorly edited fragments in the ITS2 region. The newly generated sequence has 98.81% similarity and 5 gaps (within the first 45 bases) with the holotype of *C. flavocontextus* BJTC FM2319-A (NR_191306) [20] based on the BLASTn analysis in NCBI [59]. In the present phylogenetic ITS analysis, type specimens of both *C. sinopulverulentus* and *C. flavocontextus* form one species-level clade. Vietnamese specimens initially identified as *C. flavocontextus* (LE F-344051 and LE F-344052) [18] are clustered in another clade, *Cyanoboletus* sp. 3, which also contains specimens from China and Japan. Therefore, the currently known distribution of *C. sinopulverulentus* is restricted to Shaanxi and Shanxi provinces in Northern China.

***Boletus gabretae*** Pilát, Česká Mykol. 22(3): 167 (1968)

MycoBank MB 327043.

*≡Suillellus gabretae* (Pilát) Blanco-Dios, Index Fungorum 211: 1 (2015).

*≡Cyanoboletus gabretae* (Pilát) Yang Wang, B. Zhang & Yu Li, in Wang, Ma, Wu, Yang, Liu, Rao, Dai, Gui, Tuo, Wang, Chen, Zhang & Li, Mycosphere 15(1): 925 (2024).

*Notes*: The combination of *Cyanoboletus gabretae* (Pilát) Yang Wang, B. Zhang & Yu Li, based on *B. gabretae* Pilát, although formally correct [23], lacks a solid taxonomic foundation. It is based solely on data from the literature referencing the morphochromatic character of instantaneous and intense blueing, and the presence of a yellow hymenophore. No molecular data is provided, nor any epitypification of the taxon *B. gabretae*, necessary requirements for a justified placement in the proposed genus. Moreover, the authors have not studied any specimen of this taxon [23].

Furthermore, proponents of this combination appear to ignore or contradict without any supporting scientific evidence, the statements made in the original article describing the species *B. gabretae*, which deals on a clear derivation from “*Boletus erythropus* Fr. ex Fr.” [200] (=*Boletus erythropus* Pers. s. Fries 1860, =*Neoboletus praestigiator* (R. Schulz) Svetash., Gelardi, Simonini & Vizzini [201]), on which two overlapping already known deviations would persist: (1) the overall yellow tint of every part of the basidiomes (attributed to the taxon called *Boletus junquilleus* (Quél.) Costantin & L.M. Dufour [202,203]); (2) the occasional and limited presence of a reticulum (attributed to *Boletus caucasicus* Singer, nom. inval. (=*Suillellus caucasicus* (Singer ex Alessio) Blanco-Dios, nom. inval.) [13,103,197,204,205]).

The two deviations combined would contribute to forming the entity *B. gabretae*, clearly belonging to the genus *Neoboletus* Gelardi, Simonini & Vizzini, which is reiterated to be a deviation from the well-known and widespread taxon *N. praestigiator* (“*B. erythropus* Fr. ex Fr.”) [2,13]. It should be noted that the taxon *B. gabretae* appears well understood and described even in the popular literature and on the internet (e.g., publications, forums, social media platforms), in the sense originally given to it by Pilát [200], which removes any interest in a later combination in the genus *Cyanoboletus*. However, it is currently premature to propose any combination for this taxon in *Neoboletus* due to the presence of at least two very similar species of this genus in Europe—*N. praestigiator* and *N. xanthopus*. Moreover, *B. gabretae* type material requires careful analysis, including sequencing of its genetic markers.

### 3.3. Arsenic Content

The arsenic mass fraction of 7.72 mg kg^−1^ determined in the reference material SRM 1566b matches the certified value of 7.65 ± 0.65 mg kg^−1^, indicating the quality of our analytical procedure. In the basidiome samples, the arsenic mass fractions were in the range of 0.30 to 7.14 mg kg^−1^ (Table 3), with median values of 1.56 mg kg^−1^ in *C. mediterraneensis* and 0.55 mg kg^−1^ in *C. poikilochromus*.

## 4. Discussion

Our phylogenetic analysis shows that *Cyanoboletus* forms a generic clade (multilocus: PP = 1.0, BS = 100%) with strong statistical support of all major branches (Figure 1). These results correlate with previous single-locus and multi-locus concatenated phylogenetic analyses [4,8,17,19,20,23,24,205,206]. Synonymy of *Cupreoboletus* with *Cyanoboletus* has also been confirmed.

Nuhn et al., in their LSU-based analysis, merged *Lanmaoa carminipes* (as *Boletus carminipes*) and *C. cyaneitinctus* (as *Boletus pulverulentus*) into one “carminipes” clade [207]. However, their phylogenetic reconstruction does not provide any information on generic delimitation between the later described *Cyanoboletus* and *Lanmaoa* due to limited coverage across taxa (a single species from each currently recognised genus). Other authors have expressed a more well-supported view that *Cyanoboletus* and *Lanmaoa* should be placed together in one genus due to close relationships and morphological similarities [17,206]. Although *Cyanoboletus* tends to have dull-brown colours and *Lanmaoa* often has bright-red or yellow tones [4,15,206], this is not the case with *L. fragrans*, which has a brown pileus and usually has brown tints in the stipe, especially at the base. As mentioned above, *L. fragrans* and *C. mediterraneensis* share not only similar morphological features but also often grow in the same habitats, sharing a preference for acidic soil and having many common host plants. *Cyanoboletus bessettei* and *C. fagaceophilus* (as *C. instabilis*) both share the 1/3–1/5 hymenophore-to-pileal-context ratio found in *Lanmaoa* (and some *Baorangia* species) [17,206]. Similarity between some species of *Cyanoboletus* (especially basal lineages) and *Lanmaoa* could be explained either by symplesiomorphy (inherited from the common ancestor) or homoplasy (a result of convergent evolution).

Later studies revealed two other genera, *Acyanoboletus* G. Wu & Zhu L. Yang and *Cacaoporus* Raspé & Vadthanarat, which are even more closely related to *Cyanoboletus* than *Lanmaoa* [21,198,208]. *Acyanoboletus* is the closest, characterised by a strongly incurved pileal margin when young; a pale-yellow context and hymenophore without colour changing when bruised; a stipe lacking reticulum and sometimes nearly glabrous; a strong, unpleasant smell; an intricate trichoderm pileipellis to intermediate type between trichoderm and cutis (incorrectly called “subcutis”); and subfusoid basidiospores [21,198]. *Cacaoporus* is characterised by brown to blackish-brown basidiomes, with brown encrustations in the context; chocolate-brown to dark-brown hymenophore; tubes not separable from the pileus context; white to off-white basal mycelium, which turns reddish white to pale red when bruised; amygdaliform to ovoid basidiospores; and a dark-brown spore print [208]. Therefore, both of these genera are not only clearly delimited phylogenetically from *Cyanoboletus* but also have significant morphological differences that allow a clear separation between them in the field.

Based on the current phylogenetic analysis, the genus *Cyanoboletus* has 21 phylospecies, 14 of which represent known species, and seven are undescribed. The most basal branch (PP = 1.00, BS = 97%) consists of four species-level clades split into two subclades: (a) *C. instabilis* and *C. fagaceophilus* (PP = 1.0, BS = 100%) and (b) *C. bessettei* and *Cyanoboletus* sp. 7 (PP = 1.0, BS = 100%). Species from this subspecies-level branch differ from the majority of *Cyanoboletus* species by either a much weaker blueing of tissues or remaining almost unchanging in the stipe, sometimes fading to reddish brown, as well as by a rather short hymenophore (1/3–1/5 hymenophore-to-pileal-context ratio) [15,17,21,206]. The next basal clade (PP = 1.0, BS = 100%) belongs to *C. poikilochromus*, which has been treated as a separate genus, *Cupreoboletus*, for a decade [8]. From the majority of *Cyanoboletus* spp., *C. poikilochromus* differs by the presence of a prominent reticulum on the stipe (rarely absent); minute, roundish pores, tissues that fade after blueing from copper red to copper brown; a peculiar and strong smell; numerous gloeocystidia; and needle-shaped crystals on the hymenophore and stipe surface.

The rest of the *Cyanoboletus* species belong to the crown branch (PP = 1.0, BS = 100%), which divides into two subclades: (a) *C. mediterraneensis* and *C. abieticola* (PP = 1.0, BS = 100%) and (b) the remaining *Cyanoboletus* species, including *C. pulverulentus* (PP = 1.0, BS = 100%). Species within this crown branch most closely fit the original description of *Cyanoboletus* [6] with a few additional features: (a) the hymenophore can also be subdecurrent or decurrent (*C. brunneoruber*, *C. viscidiceps*, and *Cyanoboletus* sp. 5), yellow–orange, orange, yellowish brown, brownish red to reddish brown (*C. brunneoruber*, *C. hymenoglutinosus*, and *C. macroporus*), and (b) the pileipellis can sometimes be ixotrichoderm, ixocutis, or their intermediate type (*C. abieticola*, *C. brunneoruber*, *C. hymenoglutinosus*, *C. mediterraneensis*, *C. paurianus*, *C. viscidiceps*, and *Cyanoboletus* sp. 5) [15,16,18,19,23,24].

Regarding host specificity, the vast majority of *Cyanoboletus* species grow in association with *Fagaceae* (especially *Quercus* spp.), including three Mediterranean species: *C. pulverulentus*, *C. mediterraneensis*, and *C. poikilochromus*. *Cyanoboletus mediterraneensis* also forms associations with *Cistaceae*, *Pinaceae*, and probably *Salicaceae*; *C. poikilochromus* likewise grows with those plant families and with *Betulaceae* (*Ostrya carpinifolia*). The *Cyanoboletus* species can also potentially form an ECM association with *Ericaceae* (*Arbutus unedo*), but more evidence from root tip samples is required to confirm this. *Cyanoboletus pulverulentus* has the widest range of host plant species in the entire genus, also including *Betulaceae*, *Pinaceae*, and *Malvaceae* (*Tilia*). *Cyanoboletus cyaneitinctus* forms associations with *Fagaceae* (*Quercus*) and *Juglandaceae* (*Carya*) [17,24]. *Cyanoboletus bessettei*, *C. instabilis*, and *C. macroporus* grow with both *Fagaceae* and *Pinaceae* [15,18,19]. *Cyanoboletus abieticola* is the only known species of the genus that is exclusively associated with *Pinaceae* (*Abies*) [24].

Importantly, this study has shown that spore morphology is likely a more useful taxonomic character in diagnosing *Cyanoboletus* species than previously thought. Conventional variables, such as width or length, show a significant overlap between the European species, and the chances of identifying a given collection to the species level based on these variables are often slim (Figure 5a,b). The proposed method to measure spores’ suprahilar depression, an unconventional character—variable and hard to assess with the naked eye, which we successfully demonstrate, can further aid in distinguishing European *Cyanoboletus* species (Figure 5c–f). While *C. poikilochromus* is easily identified in the field, the challenging morphological separation between *C. mediterraneensis* and *C. pulverulentus* is now clearer after detecting a pronounced suprahilar depression on the latter. We invite other researchers to apply the same method to extra-European species and expand on our species delimitation key.

Brauer et al. discovered the hyperaccumulation of arsenic, a toxic element, in *C. pulverulentus* by analysing 38 collections of this species from Europe and Macaronesia [40]. As mass fractions varied in a large range of 3.2–1300 mg kg^−1^, with a median value of 160 mg kg^−1^, and correlated with neither total nor mobile As in underlying soils [40]. In the vast majority of analysed collections, As mass fractions were higher than 50 mg kg^−1^, and lower values were very rare. The arsenic speciation in *C. pulverulentus* consisted solely of dimethylarsinic acid (DMA), and no inorganic As was detected. Because of the carcinogenic potential of DMA, *C. pulverulentus* should not be recommended as an edible mushroom [40].

In this study, we analysed five collections of *C. poikilochromus* and eight collections of *C. mediterraneensis*, with the highest detected mass fraction of 7.14 mg As kg^−1^ in the latter species. Braeuer et al. [40] similarly reported a low As value of 2.4 mg kg^−1^ for *C. cyaneitinctus* (sample ASP-82/B-28, reported as *Cyanoboletus* sp.). We therefore conclude that it is likely that none of these tested *Cyanoboletus* species hyperaccumulate As, so *C. pulverulentus* thus remains the only known As hyperaccumulator in the *Boletales* order.

## Figures and Tables

**Table 1:** The list of known *Cyanoboletus* species worldwide.

Species Name	Distribution Region	References
*C. abieticola* J. García, Ayala-Vásquez & Landeros	Central and Southern Mexico	[24]
*C. bessettei* A.R. Bessette, L.V. Kudzma & A. Farid	Southeastern USA	[17]
*C. brunneoruber* G. Wu & Zhu L. Yang	China (Yunnan)	[15]
*C. cyaneitinctus* (Murrill) A. Farid, A.R. Franck & J.A. Bolin	Canada and the USA	[14,17]
*C. fagaceophilus* G. Wu, Hai J. Li & Zhu L. Yang	Southwest and South China	[21]
*C. flavocontextus* L. Fan, N. Mao & T.Y. Zhao	China (Shanxi) and Central Vietnam	[20,22]
*C. hymenoglutinosus* D. Chakr., K. Das, A. Baghela, S.K. Singh & Dentinger	India (Sikkim)	[16]
*C. instabilis* (W. F. Chiu) G. Wu & Zhu L. Yang	Southwestern China	[15]
*C. macroporus* Sarwar, Naseer & Khalid	India (Himachal Pradesh) and Northwestern Pakistan	[15,19]
*C. mediterraneensis* Biketova, Rinaldi & Simonini	Southern Europe and Israel	[10,13]
*C. paurianus* K. Das & A. Gosh	India (Uttarakhand)	[19]
*C. poikilochromus* (Pöder, Cetto & Zuccherelli) M. Carbone, D. Puddu & P. Alvarado	Southern Europe and the Levant	[8,9,13,14]
*C. pulverulentus* (Opat.) Gelardi, Vizzini & Simonini	Europe, Asia, Australia, North Africa, North America, and Colombia	[9,12,13,14]
*C. sinopulverulentus* (Gelardi & Vizzini) Gelardi, Vizzini & Simonini	Western China and India	[3,15,19]
*C. viscidiceps* Yang Wang, G. Rao, B. Zhang & Y. Li	China (Jilin)	[23]

**Table 2:** A summary of the characteristics of each DNA sequence alignment used for phylogenetic inference in the present work.

Analysis	No. of Aligned Sequences	No. of Collections	Alignment Length (bp)	No. of Variable Sites	No. of Parsimony Informative Sites	No. of Singleton Sites
Multi-locus	285	149	3051	961	762	195
ITS	115	114	793	365	284	80
LSU	67	66	853	148	114	34
*tef1*-α	48	47	624	213	166	47
*rpb2*	55	54	779	235	198	34

**Table 3:** Arsenic content in dry basidiomes of *Cyanoboletus mediterraneensis* and *C. poikilochromus*.

Material	Origin	Fungarium Sample	As (mg kg^−1^)
*C. mediterraneensis*	Greece	ACAM 2022-134	1.33
	Israel	K-M001443116	3.39
	Israel	K-M001443117	4.67
	Italy	ACR-Hal-BP-25	2.74
	Italy	K-M001445821	0.30
	Italy	GS10270	1.43
	Spain	VAL_Myco 1758	1.68
	Spain	VAL_Myco 1757	0.72
*C. poikilochromus*	Israel	K-M001441521	0.34
	Israel	K-M001441523	7.14
	Israel	K-M001441529	0.55
	Spain	VAL_Myco 1755	0.36
	Spain	VAL_Myco 1756	0.55
Reference material			
NIST SRM 1566b			7.72

## Data Availability

The original contributions presented in this study are included in the article and Supplementary Materials. Also, some data can be found in publicly available datasets at https://www.ncbi.nlm.nih.gov/ and http://www.mycobank.org/, accessed on 13 March 2026. Further inquiries can be directed to the corresponding authors.

## Supplementary Materials

The following supporting information can be downloaded at: https://www.mdpi.com/article/10.3390/jof12050315/s1. File S1: List of examined material. File S2: Aligned and concatenated dataset of *Cyanoboletus* and closely related genera (ITS, nrLSU, *tef1-α*, *rpb2*). Table S1: Spore size statistics of *Cyanoboletus* spp. from the Mediterranean Basin. Table S2: Information on specimens used in multilocus phylogenetic analysis and their GenBank accession numbers. Newly generated sequences are in boldface. Sequences submitted in public repositories, but not used in phylogeny, are marked with an asterisk (*). Table S3: Substitution models and partition schemes used in multi- and single-locus phylogenetic analyses.
