# Supplementary material for: Revision of the Genus Cyanoboletus (Boletaceae) in the Mediterranean Basin with Notes on Arsenic Hyperaccumulation
Source: J Fungi (Basel). 2026 Apr 25;12(5):315. doi: 10.3390/jof12050315 (PMC13208421; doi:10.3390/jof12050315)
Supplement: Supplementary file 1 [file jof-12-00315-s001.zip › Supplementary File S1.pdf]

S1. List of Examined Material

Cyanoboletus pulverulentus **(Opat.) Gelardi, Vizzini & Simonini**
BULGARIA, Sofia Province: Plana Mt., between the mineral springs at Zheleznitsa village and Krastatia Dab landmark, under *Quercus* sp. on siliceous soil, 25.08.2002, leg. B. Assyov, SOMF 25394; Sofia city, Vrana Park, 442°38'20.5"N 23°25'48.3"E, 575 m, in a landscape forest with *Tilia* sp. and *Abies* sp., on siliceous soil, 23.07.2018, leg. B. Assyov, SOMF 30990; Vitosha Mt., above Bistritsa village, under *F. sylvatica*, on siliceous soil, 14.08.2002, leg. B. Assyov, SOMF 25393; ibid., 03.09.2002, leg. B. Assyov, SOMF 27159 and SOMF 27160; ibid., 31.07.2004, leg. B. Assyov, SOMF 27161, SOMF 27162, and SOMF 27163;
CZEKHIA, Central Bohemia: Čerčany, 49°50'42.5"N 14°41'57.1"E, with *Tilia cordata* and *Quercus robur*, 10.06.2016, J. Borovička, PRM 944001, GenBank: ITS – LT714706; Prague, not far from Čínský pavilon, 50°04'02.6"N 14°21'16.0"E, with *Q. robur* and *C. betulus*, 09.06.2016, leg. J. Borovička, PRM 944014, GenBank: ITS – LT714705; Rakovník, Jesenice, 50°05'23.1"N, 13°29'11.0"E, in mixed forest plantation under *Q. robur* and *Corylus avellana*, 17.06.2016, leg. J. Borovička, PRM 944013 (epitype), GenBank: ITS – LT714707; Highland: Úsobí, Humpolec, 49°30'46.5"N 15°30'04.2"E, with *T. cordata*, 19.06.2016, leg. F. Zíka, PRM 944022, GenBank: ITS – LT714708; South Bohemia: Jindřichův Hradec, 49°08'46.5"N 14°59'56.3"E, with T. cordata, 08.06.2016, leg. J. Borovička, PRM 935997, GenBank: ITS – LT714709;
HUNGARY, Borsod-Abaúj-Zemplén Co.: Zemplén Mt., Mt. Nagy-Milic, near Hollohaza Laszlotanya, 48°34'29.8"N 21°26'17.9"E, *F. sylvatica* forest, 28.07.2018, leg. A. Yu. Biketova & B. Bálint, K-M001445829 (AB B18-391), GenBank: ITS – PZ244190; ibid., 48°34'30"N 21°26'18"E, *F. sylvatica* forest, 28.07.2018, leg. T. Varga & A. Yu. Biketova, K-M001445827 (AB B18-412); Nógrád Co.: Diósjenő, 47°57'06"N 18°59'21"E, *F. sylvatica* forest, 24.06.2018, leg. L. Albert, B. Dima & A. Yu. Biketova, K-M001445822 (AB B18-392), GenBank: ITS – PZ244189;
ITALY, Emilia-Romagna: Parma (PR), Parco Ducale di Parma, 44°48'23"N 10°19'10"E, 72 m, under *Tilia* sp., 09.10.2015, leg. G. Simonini, GS10798; Gattatico (RE), Praticello, Asilo Girasole, 44°48'24"N 10°28'23"E, 37 m, with *Tilia* sp., 05.10.1991, leg. G. Donelli, GS836; Villa Minozzo (RE), Camping Febbio 2000, 44°17'46"N 10°25'52"E, 1158 m, with *Fagus sylvatica*, 24.08.1997, leg. G. Simonini, MCVE 18188 (GS1826), GenBank: ITS – JF907794; Lazio: Leonessa (RI), Villa Pulcini, 42°60'34″N 12°90'43″E, 955 m, with *C. avellana*, *Q. pubescens*, *Q. cerris*, *C. betulus* and *F. sylvatica*, 29.07.2014, leg. M. Gelardi & F. di Rita, MG628, GenBank: ITS – KT157055, LSU – KT157064, *tef1-α* – KT157073, *rpb2* – KY157069; Lombardy: Cevo (BS), Valsaviore, 46°09'02″N, 10°40'96″E, 1240 m, on acidic soil with *C. avellana*, 16.08.2008, leg. M. Gelardi, MG126, GenBank: ITS – KT157053, LSU – KT157062; Sardinia: Iglesias (SU), Bellicai, 39°19'04" N 08°30'14" E, 280 m, under *Q. suber*, 15.10.2022, leg. M. Scano, GS11209; Sicily: Zafferana Etnea (CT), Mt. Etna, Contrada Ilice di Carrinu, Caselle, 37°43'04" N, 15°05'29" E, 921 m, with *Q. ilex*, *Q. pubescens*, *Acer campestre*, and *O. carpinifolia*, 08.11.2016, leg. G. Simonini, GS10910, GenBank: ITS – PZ244186; ibid., 37°43'06" N, 15°05'43" E, 955 m, with *Q. ilex*, *Q. pubescens*, *Acer campestre*, and *O. carpinifolia*, 08.11.2016, leg. G. Simonini, GS10909; Toscana: Sorano (GR), left bank of the Lente stream, 42°41'04" N, 11°42'45" E, 300 m, with *C. avellena*, *Q.cerris*, and *C.sativa*, 26.10.2022, leg. G. Simonini, GS11186, GenBank: ITS – PZ244187; unknown locality, found at a fungi exhibition, 26.09.2014, leg. anonymous, GS10257, GenBank: ITS – PZ244185;
PORTUGAL, Azores: Flores island, Porto da Lomba by rocky shore, 39°24@45″N 31°9@3.2″W, among moss, under *Pinus* sp., 27.09.2022, leg. J. Lucas & V. Fachada, PO-F2601, GenBank: ITS – PV461258; São Miguel, Miradouro da Ponta do Sossego, 37°47'55.9"N 25°08'48.2"W, under planted *P. nigra* trees, 09.10.2011, leg. T. Lezzi, MG456, GenBank: ITS – KT157054, LSU – KT157063; Madeira: Ribeiro Frio, 32°43'58.4"N, 16°53'21.2"W, with *Q. robur*, 26.09.2015, leg. J. Borovička, PRM 935923, GenBank: ITS – LT714704;

SWITZERLAND, canton of Zurich: Wangen-Brüttisellen, park in the town, 47°26'00"N 8°35'53"E, 484 m, with *P. abies* and *F. sylvatica*, on the ground, among *Polytrichum*, 28.07.1991, leg. C. Lavorato, GS1551;
UNITED KINGDOM, England: London, Richmond, Royal Botanic Gardens, Kew, near Princess Walk trail, 51°28′49″N, 00°17′44″W, 10 m, among grass, near *Quercus* sp., 09.11.2021, leg. MSc students & A. Yu. Biketova, K-M001445690 (AB B21-386), GenBank: ITS – PZ244188; London, Richmond, Royal Botanic Gardens, Kew, on the right side of a track running toward river Thames, between the southern shore of Lake Crossing and the Arboretum Nursery, 10 m, under *Tsuga* spp., 10.10.2025, leg. M. Gelardi, MG1050 (GP); Wales: Aberdare, Brecon Beacons National Park, on open area, next to the tourist path, 51°47'48.1"N 3°33'38.8"W, with *C. avellana*, 09.08.2025, leg. A. Yu. Biketova, K-M001448123 (AB W5), GenBank: ITS – PZ244184.

***Cyanoboletus mediterraneensis* f. *mediterraneensis* Biketova, Rinaldi & Simonini**
BULGARIA: Varna Province: Dolni Chiflik municipality, between Novo Oryahovo and Shkorpilovtsi villages, 42°59'28.4"N 27°53'06.8"E, 5 m, coastal forests dominated by *Q. robur*, mixed with *Q. cerris*, *Acer campestre*, and *C. orientalis*, on sandy and possibly with some lime content soil (wooded dunes), 24.09.2024, leg. B. Assyov, SOMF 30989, GenBank: ITS – PZ244175;
GREECE, Crete: Kourna Lake, solitary mature basidiome, in a tree stand under *Q. coccifera*, on calcareous soil, 19.10.2022, leg. G.I. Zervakis, ACAM 2022-134, GenBank: ITS – OR770584, LSU – PZ231930; Epirus: Ioannina City, several middle-aged to mature basidiomes, in a suburban forest, under *Q. ilex*, 22.10.2013, leg. G. Konstantinidis, GK6821, GenBank: ITS – PZ244162;
ISRAEL, Carmel Mount (Haifa District): Mt. Carmel National Park, Nahal Nesher, solitary basidiome under *P. halepensis*, 01.02.2004, leg. T. Pavlíček & S.P. Wasser, K-M001443117 (AB B04-01), GenBank: ITS+LSU – PZ239095; Ofer, Nahal Timon Forest, 32°37'01.6"N, 34°59'41.0"E, 143 m, solitary basidiome growing with *Q. calliprinos*, 17.12.2022, leg. O. Skusky, K-M001445227 (AB B22-401), GenBank: ITS – PZ244174; Samaria (Haifa District): Ramat Hanadiv Park, solitary basidiome under *Q. calliprinos*, 08.11.2006, leg. Y. Ur, K-M000265124 (ex herb. HAI A-001; paratype), GenBank: ITS – OM801199; Sharon Plain (Haifa District): Alona forest, solitary basidiome under *Q. calliprinos*, 06.12.2024, leg. Yu. Vilozni, K-M001445825 (AB B24-424); near Zikhron Ya'akov, 32°34'N, 34°58'E, four mature basidiomes in *Q. calliprinos* forest, 19.12.2017, leg. A. Krishtul & R. Kuznetsov, AB B17-324 (lost specimen), GenBank: ITS+LSU – PZ239094; Upper Galilee (Northern District): Abirim, in a spot where a yard borders with a natural forest, two mature and two immature caespitose basidiomes, on the ground near the trunk of *Q. calliprinos*, 33°2'28"N, 35°17'26" E, 575 m, 25.07.2020, leg. N. Gabdank & Y. Segal, AB B20-372 (lost specimen); Goren Park, solitary basidiome under Q. calliprinos, 01.12.2012, leg. Z. Shafranov & A. Yu. Biketova, det. A. Yu. Biketova, K-M000265123 (ex herb. HAI B12-077; holotype), GenBank: ITS – PZ244171, LSU – NG_228932; ibid., solitary basidiome under *Pistacia* sp. in *Q. calliprinos* forest, 05.12.2016, leg. Z. Shafranov, S. Shafranov & E. Shafranov, K-M000265125 (AB B15-279; paratype), GenBank: ITS – PZ244172; close to the previous locality, 33°03'15.7"N, 35°13'43.0"E, solitary basidiome under *Q. calliprinos*, 31.12.2022, leg. Z. Shafranov, K-M001443116 (AB B22-400), GenBank: ITS – PZ244173;
ITALY, Apulia: Tricase (LE) suburbs, Mr. Gerardo's private garden, 39°55'49"N 18°21'31"E, 102 m, with *Q. ilex*, 01.11.2012, leg. A. Errico, GS10265, GenBank: ITS – PZ244165; Tricase (LE) suburbs, 39°55'09"N 18°22'22"E, 100 m, with *Q. cerris* and *Q. coccifera*, 02.11.2012, leg. A. Errico, K-M001445823 (GS10270); Emilia-Romagna: Castelfranco Emilia (MO), Bosco Albergati, la Cavazzona, 44°34'53"N 11°07'55"E, 39 m, under *Q. robur*, 22.10.2012, leg. A. Testoni, GS10098, GenBank: ITS – PZ244163; Lazio: Ostia (RM), Castelfusano, next to Via

Cristoforo Colombo roadside, 41°42'58.0''N 12°19'12.3''E, 2 m, in a termophilic lowland mixed Mediterranean woodland close to seaside, under *Q. ilex* with the presence of *P. pinea*, *Arbutus unedo*, *Pistacia lentiscus*, *C. salvifolius*, *Phillyrea latifolia*, and *Erica arborea*, on sandy calcareous soil, 08.10.2022, leg. F. Costanzo, M. Gelardi and O. Gelardi, MG948; Roma, Appia Antica Archaeological Park, 41°50'12.4''N 12°32'20.6''E, 56 m, in the garden of a private property, under *Q. ilex* with the presence of *Cupressus sempervirens*, on loamy acidic soil, 25.09.2009, leg. M. Gelardi, MG237; ibid., four mature specimens, 23.10.2015, leg. M. Gelardi, MG689; ibid., four middle-aged to mature specimens and two primordia, 22.09.2021, leg. M. Gelardi, MG870; Lombardy: Carbonara al Ticino (PV), strada per Carbonara, left bank of the Ticino river, 45°10'03''N 09°03'12''E, 83 m, under *Q. ruber*, 29.08.2022, leg. C. Veneroni, GS11189; Carbonara al Ticino (PV), Canarazzo, 45°11'38''N 09°05'10''E, 60 m, with *Q. robur*, 30.09.2022, leg. C. Veneroni, GS11161, GP; Carbonara di Ticino (PV), left bank of Ticino river, 45°10'04''N 9°03'11''E, 78 m, with *Q. robur*, 02.09.2022, leg. G. Simonini, GS11190; Castelleone (CR), Luna Bassa, Regona, 45°16'18''N 09°46'27''E, 56 m, with *Q. robur*, 07.10.2021, leg. M. C. Morosini, GS11222, GenBank: ITS – PZ244168; Castelleone (CR), Parco Becchetto, Piazza Fepenica, 45°17'48''N 09°46'18''E, 64 m, with *Q. robur* and *Acer pseudoplatanus*, 10.09.2022, M. C. Morosini, GS11225, GenBank: ITS – PZ244169; Genivolta (CR), Tombe Morte, 45°19'54'' N, 09°51'35'' E, 73 m, with *Q. robur*, 31.08.2022, leg. M. C. Morosini, GS11221, GenBank: ITS – PZ244167; Sardinia: Assemini, two mature basidiomes under a *Q. ilex* tree, in an urban park, 12.7.2022, leg. M. Scano, ACR-2024-4-MS-1, GenBank: ITS – PP938742; Gonnesa, two mature basidiomes in pure *H. halimifolium* shrubland, 26.10.2015, leg. A.C. Rinaldi, ACR-Hal-BP-25 (paratype), GenBank: ITS – MT594497; Pixinortu, San Sperate, a single basidiome in a mixed *Quercus* and *Populus* stand, 12.6.2023, leg. M. Scano, ACR-2024-5-MS-2, GenBank: ITS – PP938743; Sicily: Casarò (ME), Portella dei Bufali, 37°52'15''N 14°41'17''E, 1180 m, with *Q. cerris* and *Quercus* sp., 21.10.2013, leg. G. Vasquez, K-M001445821 (GS10115), GenBank: ITS – PZ244164; Monterosso Almo (RG), Parco Forestale Canalazzo, Contrada Canalazzo, 37°05'18''N 14°43'59''E, 567 m, with *Q. ilex* and *Q. pubescens*, 09.11.2016, leg. G. Vasquez, GS10927, GenBank: ITS – PZ244166;

PORTUGAL, Lisboa e Vale do Tejo: Sesimbra, Alto das Vinhas, 38°29[illegible]36″N, 9°3[illegible]56.8″W, 110 m, in sandy *Q. suber* montado forest, 17.10.2022, leg. V. Fachada, PO-F2442, GenBank: ITS – PV170925;

SPAIN, Valencian Community: Alicante, Tollos, La Foia Roja, Masos de Capaimona, 38°46'22.14''N, 0°15'4.64''W, 743 m, under *Q. rotundifolia*, 21.10.2015, leg. A. Conca, ACM-215059; ibid., 21.09.2023, leg. I. Garrido-Benavent, VAL_Myco 1757 (IGB1682), GenBank: ITS – PZ244170; Valencia, Devesa de l'Albufera, Garrofera firewall, 39°21'19.18''N, 0°19'22.47''W, 4 m, in a mixed forest with *P. pinea*, *P. halepensis*, *Q. coccifera* and *Myrtus communis*, 28.09.2018, leg. A. Conca, ACM-218045; ibid., 31.10.2022, leg. A. Conca, VAL_Myco 1758 (ACM-222080), GenBank: ITS – PZ244161.

***Cyanoboletus mediterraneensis* f. *pallidus* Angeli, Baldazzi, Gelardi & Biketova**

ITALY, Marche: Rimini, Villa Verrucchio, Via Farneto 25, in a private garden, 43°59'59.3''N 12°26'39.9''E, 126 m, three basidiomes growing with *Salix* sp., *Rosa* hybrid, *Jasminum* sp., *Quercus* sp. (30 m away), 13.11.2025, leg. P. Angeli & L. Baldazzi, det. M. Gelardi, MCVE 31989 (collector's number PAn1213_13112025; holotype), GenBank: ITS – PZ244160.

***Cyanoboletus poikilochromus* (Pöder, Cetto & Zuccher.) M. Carbone, D. Puddu & P. Alvarado**

BULGARIA, Haskovo Province: Svilengrad municipality, between Shtit and Raykova Mogila villages, 41°49'19.0''N 26°19'40.5''E, 185 m, under *Q. pubescens* on calcareous soil, 02.10.2014, leg. B. Assyov & N. Apostolov, SOMF 30350 , GenBank: ITS – OL774786;

GREECE, North Aegean: Lesbos, Amali, with *P. brutia* and *Q. ithaburensis* ssp. *macrolepis*, 19.10.2010, leg. A. Sgatzos, GK 5275; Ionian Islands: Corfu, Sinies, under *Q. ilex*, 21.10.2023, leg. G. Konstantinidis, GK 15749;
FRANCE, Provence-Alpes-Côte d'Azur: Alpes-Maritimes, Biot, Brague Departmental Nature Park, under *Q. ilex*, 15.10.2007, leg. S.E. Evans, K-M000156177;
ISRAEL, Carmel Mount (Haifa District): Mt. Carmel National Park, Dereh Nof HaCarmel, on ground under *Q. calliprinos*, 17.11.2012, leg. O. Godorova & Z. Shafranov, K-M001441527 (AB B12-031); Mt. Carmel National Park, near Beit Oren, Henyon Ha'Agam, under *Q. calliprinos*, 10.11.2012, leg. Z. Shafranov, K-M001441525 (AB B12-007); Mt. Carmel National Park, Dereh Nof HaCarmel, under *Q. calliprinos*, 18.11.2012, leg. O. Godorova, K-M001441529 (AB B12-070), GenBank: ITS – PZ244182; Mt. Carmel National Park, Henyon Ha'Agam, under *Q. calliprinos*, 27.10.2012, leg. Z. Shafranov, K-M001441528 (AB B12-168); Mt. Carmel National Park, near the crossroad Damon, 32°44'03''N 35°02'22''E, 505 m, under *Q. calliprinos*, 30.10.2015, leg. O. Copel & E. Kalichman, K-M001441523 (AB B15-262); Lower Galilee (Northern District): near Yodfat Junction, under *Q. calliprinos*, 22.11.2006, leg. Y. Ur, K-M001441522 (ex heb. HAI A-063), GenBank: ITS – PZ244181; Upper Galilee (Northern District): Hanita Forest, on ground in mixed forest of *Q. calliprinos* and *P. halepensis*, 06.10.2011, leg. Y. Cherniavsky, K-M001441531 (AB B11-02), GenBank: ITS – PZ244183, LSU – PZ231927; Goren Park, under *Q. calliprinos*, 29.11.2012, leg. A. Rotenberg, K-M001441521 (AB B12-085), GenBank: ITS – PZ244180, LSU – PZ231928; Mt. Meron, under *Q. calliprinos*, 08.11.2014, leg. A. Rotenberg, K-M001441524 (AB B14-200); Mt. Meron, under *Q. calliprinos*, 07.11.2015, leg. R. Kuznetsov & Z. Shafranov, K-M001441520 (AB B15-272);
ITALY, Emilia Romagna: Ravenna, Pineta di Classe, 44°21′31″N, 12°16′49″E, 5 m, with *P. pinea*, *Q. robur*, *Q. ilex*, and *Crataegus* sp., 10.09.1981, leg. A. Zuccherelli, det. A. Zuccherelli & R. Pöder, IB 19810625 (holotype); ibid., 10.09.1987, leg. A. Zuccherelli, TO HG10091987 (epitype), GenBank: ITS – KT157047, LSU – KT157056; Ravenna, Fassatone, Pineto bei Vitola, near *Q. ilex*, 05.09.1996, leg. A. Zuccherelli, IB 19960585 (paratype), GenBank: ITS – PZ244176, LSU – PZ231929; Reggio nell'Emilia, Quattro Castella, Parco di Roncolo, 44°37'20''N 10°29'23''E, 320 m, with *Q. pubescens* and *O. carpinifolia*, 29.09.2000, leg. G. Simonini, GS10070, GenBank: ITS – KT157051, LSU – KT157060, *tef1-α* – KT157072, *rpb2* – KT157068; ibid., 28.09.2014, leg. G. Simonini, GS10171 and GS10172; ibid., 20.10.2010, leg. G. Simonini, GS11008, GenBank: ITS – KT157050, LSU – KT157059, *tef1-α* – KT157071, *rpb2* – KT157067; ibid., 44°37'32'' N, 10°29'10''E, 330 m, with *Q. pubescens*, 14.10.1991, G. Simonini, GS855; ibid., 360 m, on calcareous soil, with *Q. pubescens* and *O. carpinifolia*, 09.10.2010, leg. M. Gelardi & G. Simonini, MG367, GenBank: ITS – KT157049, LSU – KT157058; Quattro Castella (RE), Bergonzano, 44°37'22''N 10°28'27''E, 365 m, with *Q. pubescens*, 15.09.1986, leg. G. Simonini, GS316; ibid., 19.09.1993, leg. G. Simonini, GS995; ibid., 44°37'24''N, 10°28'27''E, 380 m, 06.10.1991, leg. G. Simonini, GS839; Traversetolo (PR), urban flowerbed, 44°38'38''N, 10°22'35''E, 176 m, under *Tilia* sp., 14.09.2018, leg. E. Ponzi, GS11063; Vetto (RE), Strada Costa on the border of the 1st hairpin bend, 44°29'15''N 10°20'32''E, 533 m, *Q. pubescens*, 13.09.1982, leg. G. Simonini, GS63; Lazio: Rome, Fregene, Oasi di Macchiagrande, 41°49'46''N 12°12'50''E, 15 m, on sandy, calcareous soil with *Q. ilex*, *Q. robur*, *A. unedo* and *E. arborea*, 20.10.2009, leg. G. Minniti, M. Gelardi & V. Migliozzi, MCVE 25603 (MG271), GenBank: ITS – KT157048, LSU – KT157057, *tef1-α* – KT157070; ibid., Fregene, Pineta Monumentale di Fregene, urban public park, 15 m, under *Q. ilex* with the presence of *P. pinea*, 28.10.2023, leg. F. Costanzo, M. Gelardi & O. Gelardi, MG1004;
SPAIN, Valencian Community: Alicante prov., Vall de Gallinera, Corral de Pere en Jordi, 38°50'47.66''N, 0°15'13.09''W, 679 m, under *Q. rotundifolia*, 17.09.2023, leg. I. Garrido-Benavent, VAL_Myco 1755 (IGB1667), GenBank: ITS – PZ244178; ibid., 38°51'10.46''N 0°15'25.75''W, 796 m, under *Q. rotundifolia*, 17.09.2023, leg. I. Garrido-Benavent, VAL_Myco 1756 (IGB1673), GenBank: ITS – PZ244179; Valencia prov., Valencia, Barx, Barranc de Manesa, 39°0'6.16''N

0°18'52.74"W, 518 m, under *Q. rotundifolia*, 16.10.2022, leg. I. Garrido-Benavent, VAL_Myco 1768 (IGB1422), GenBank: ITS – PZ244177.

***Cyanoboletus sinopulverulentus* (Gelardi & Vizzini) Gelardi, Vizzini & Simonini**
CHINA, Shaanxi Province: Qinling Mountains, Heihe National Natural Forest Park, Yingbanliang village, 1432 m, on very moist and drained soil under *Castanea mollissima*, 30.09.2011, leg. M. Gelardi & J.-Z. Sun, HMAS 266894 (isotypes: TO HG2821, MG434), GenBank: ITS – PZ244192.
