# Supplementary material for: Revision of the Genus Cyanoboletus (Boletaceae) in the Mediterranean Basin with Notes on Arsenic Hyperaccumulation
Source: J Fungi (Basel). 2026 Apr 25;12(5):315. doi: 10.3390/jof12050315 (PMC13208421; doi:10.3390/jof12050315)
Supplement: Supplementary file 1 [file jof-12-00315-s001.zip › Supplementary Tables S2 and S3.pdf]

Table S2. Information on specimens used in multilocus phylogenetic analysis and their GenBank accession numbers. Newly generated sequences are in boldface. Sequences submitted in public repositories, but not used in phylogeny, are marked with an asterisk (*).

Species	Voucher	Geographic origin	GenBank Accession Number				Notes
			ITS	LSU	*tef1-α*	*rpb2*	
Cyanoboletus abieticola	MEXU-30109	Mexico: Oaxaca	MW209738	MW750368	-	PP108650	Holotype
C. abieticola	ITCV-1010	Mexico: Oaxaca	-	MW750367	-	-	Paratype
C. abieticola	MEXU-30111	Mexico: Oaxaca	MW209739	MW750366	-	PP108649	Paratype
C. abieticola	MEXU-30106	Mexico: Oaxaca	MW209740	MW750365	-	-	Paratype
C. abieticola	MUAQ13	Mexico: Querétaro	MW750332	MW750369	-	-	Paratype
C. abieticola	MEXU-26275	Mexico: Tlaxcala	KC152077	-	-	-	Paratype
C. abieticola	MEXU-26276	Mexico: Tlaxcala	KC152076	-	-	-	Paratype
C. abieticola	MEXU-26278	Mexico: Tlaxcala	KC152075	-	-	-	Paratype
C. bessettei	USF 301500 (A)	USA	MW675737	MW662571	MW737482	MW737457	Holotype
C. bessettei	ARB1393B	USA	MW675738	-	MW737483	MW737458	Paratype
C. bessettei	iNaturalist 180267242 (OMDL K. Canan)	USA: North Carolina	PP156444	-	-	-	-
C. bessettei	iNaturalist 13923264 (S.D. Russell NAMA2018 MS)	USA: Mississippi	OP541725	-	-	-	-
C. bessettei	DUKE:0352590	USA: South Carolina	OL342390	-	-	-	-
C. bessettei	DUKE:0351605	USA: South Carolina	OL342399	-	-	-	-
C. brunneoruber	HKAS 80579-1 (HKAS 80579 copy 1)	China: Yunnan	-	KT990568	KT990763	KT990401	Holotype
C. brunneoruber	HKAS 80579-2 (HKAS 80579 copy 2)	China: Yunnan	-	KT990569	KT990764	KT990402	Holotype
C. brunneoruber	HKAS 50347	China: Yunnan	-	KF739692	KF739806	KF739730	Paratype
C. brunneoruber	HKAS 52558	China: Yunnan	-	KF739691	KF739805	KF739729	Paratype
C. brunneoruber	HKAS 63504	China: Yunnan	-	KF112368	KF112194	KF112702	Paratype
C. brunneoruber	OR0233	China	-	-	MG212586	MG212628	-
C. cyaneitinctus	PRM 944518	USA: New York	LT714710	MF373585	-	-	-
C. cyaneitinctus	USF 288424	USA	MW675739	MW662574	-	MW737461	-
C. cyaneitinctus	USF 301499	USA	MW675744	MW662579	-	MW737503	Epitype
C. cyaneitinctus	JAB184	USA	MW675731	MW662584	-	MW737467	-
C. cyaneitinctus	JAB324	USA	MW675732	MW662586	-	MW737469	-
C. cyaneitinctus	JAB325	USA	MW675733	-	-	MW737470	-
C. cyaneitinctus f. *reticulatus*	Farid 1035	USA	MZ746113	-	-	-	-
C. cyaneitinctus	SR06T3-171	USA: Ohio	FM999526	-	-	-	-
C. cyaneitinctus	iNaturalist 32191013	USA: Iowa	ON534167	-	-	-	-

C. cyaneitinctus	iNaturalist 184805641 (OMDL10)	USA: Kentucky	PP850729	-	-	-	-
C. cyaneitinctus	Mushroom Observer 323745	USA: New Jersey	ON705304	-	-	-	-
C. cyaneitinctus (as *C. pulverulentus*)	9606	USA: Massachusetts	-	KF030313	KF030418	-	-
C. cyaneitinctus (as Uncultured *Basidiomycota*)	man24_soil_H05	USA: Michigan	GU328532	-	-	-	Environmental
C. cyaneitinctus	S.D. Russell ONT (Mushroom Observer 496468)	USA: Pennsylvania	OP749305	-	-	-	-
C. cyaneitinctus	MICH139837	USA: Michigan	OM985877	-	-	-	-
C. cyaneitinctus	RT00004	USA: Wisconsin	EU819502	-	-	-	-
C. cyaneitinctus (as *C. pulverulentus*)	MICH KUO-09220408	USA: Wisconsin	-	MK601732	MK721086	MK766294	-
C. cyaneitinctus	iNaturalist 55864593	USA: New York	MT939495	-	-	-	-
C. cyaneitinctus	JMP0012	USA: Wisconsin	EU819453	-	-	-	-
C. cyaneitinctus	iNaturalist 66898219	Canada: Quebec	ON943338	-	-	-	-
C. cyaneitinctus	B1915	Canada	KY826057	-	-	-	-
C. fagaceophilus	HKAS 126556	China	NR_189954	NG_229119	OQ873455	OQ873494	Holotype
C. fagaceophilus	HKAS 80691	China	-	-	OQ873456	OQ873495	-
C. fagaceophilus	HKAS 123872	China	-	OQ888717	OQ873454	OQ873493	-
C. fagaceophilus (as *C. instabilis*)	FHMU1839 (N.K.Zeng2862	China: Yunnan	MG030473	MG030466	MG030478	-	-
C. hymenoglutinosus	CAL DC 14-010	India	NR_164239	KT860060	-	-	Holotype
C. instabilis	HKAS 59554	China: Yunnan	-	KF112412	KF112186	KF112698	-
C. macroporus	DC 21-02	India: Himachal Pradesh	OQ860238	OQ860239	-	ON364552	-
C. macroporus	DC 21-24	India: Himachal Pradesh	OQ860240	OQ860241	-	OQ876894	-
C. macroporus	LAH35252	Pakistan: Khyber Pakhtunkhwa	MW045557	-	-	-	Holotype
C. macroporus	LCWH12011 (sarwar1)	Pakistan: Khyber Pakhtunkhwa	MW369503	-	-	-	Paratype
C. mediterraneensis	**SOMF 30989**	**Bulgaria: Varna Prov.**	**PZ244175***	-	-	-	-
C. mediterraneensis	**ACAM 2022-134**	**Greece: Crete**	**OR770584**	**PZ231930**	-	-	-
C. mediterraneensis	**GK6821**	**Greece: Epirus**	**PZ244162**	-	-	-	-
C. mediterraneensis	**K-M001443117 (AB B04-01)**	**Israel: Carmel Mount**	**PZ239095**		**-**	**-**	**-**
C. mediterraneensis	**AB B17-324**	**Israel: Sharon Plain**	**PZ239094**		**-**	**-**	**-**
C. mediterraneensis	**K-M001445227 (AB B22-401)**	**Israel: Sharon Plain**	**PZ244174**	**-**	**-**	**-**	**-**

C. mediterraneensis	**K-M000265123 (ex herb. HAI B12-077)**	**Israel: Upper Galilee**	**PZ244171**	NG_228932	**-**	**-**	**Holotype**
C. mediterraneensis	**K-M000265125 (AB B15-279)**	**Israel: Upper Galilee**	**PZ244172**	**-**	**-**	**-**	**Paratype**
C. mediterraneensis	**K-M001443116 (AB B22-400)**	**Israel: Upper Galilee**	**PZ244173**	**-**	**-**	**-**	**-**
C. mediterraneensis	**GS10098**	**Italy: Emilia-Romagna**	**PZ244163**	-	-	-	-
C. mediterraneensis	**K-M001445821 (GS10115)**	**Italy: Sicily**	**PZ244164**	-	-	-	-
C. mediterraneensis	**GS10265**	**Italy: Apulia**	**PZ244165**	-	-	-	-
C. mediterraneensis	**GS10927**	**Italy: Sicily**	**PZ244166**	-	-	-	-
C. mediterraneensis	**GS11221**	**Italy: Lombardy**	**PZ244167**	-	-	-	-
C. mediterraneensis	**GS11222**	**Italy: Lombardy**	**PZ244168**	-	-	-	-
C. mediterraneensis	**GS11225**	**Italy: Lombardy**	**PZ244169**	-	-	-	-
C. mediterraneensis	**ACR-2024-4-MS-1**	**Italy: Sardinia**	**PP938742**	-	-	-	-
C. mediterraneensis	**ACR-2024-5-MS-2**	**Italy: Sardinia**	**PP938743**	-	-	-	-
C. mediterraneensis	**PO-F2442**	**Portugal: Sesimbra**	**PV170925***	**-**	**-**	**-**	**-**
C. mediterraneensis	**VAL_Myco 1757 (IGB1682)**	**Spain: Valencian Com.**	**PZ244170**	-	-	-	-
C. mediterraneensis	**VAL_Myco 1758 (ACM-222080)**	**Spain: Valencian Com.**	**PZ244161**	-	-	-	-
C. mediterraneensis	K-M000265124 (ex herb. HAI A-001)	Israel: Samaria	OM801199	-	-	-	Paratype
C. mediterraneensis	TUR-A 209100	Italy: Ligury	MZ265180	MZ265195	MZ277235	MZ277225	-
C. mediterraneensis	TUR-A 209198	Italy: Ligury	MZ265182	MZ265197	MZ277237	MZ277227	-
C. mediterraneensis	TUR-A 208928	Italy: Ligury	MZ265179	MZ265194	MZ277234	MZ277224	-
C. mediterraneensis	TUR-A 209199	Italy: Ligury	MZ265183	MZ265198	MZ277238	MZ277228	-
C. mediterraneensis	ACR-Hal-BP-25	Italy: Sardinia	MT594497	-	-	-	Paratype
C. mediterraneensis	TUR-A 208929	Italy: Sardinia	MZ265181	MZ265196	MZ277236	MZ277226	-
C. mediterraneensis* f. *pallidus	**MCVE 31989 (PAn1213_131 12025)**	**Italy: Marche**	**PZ244160***	**-**	**-**	**-**	**Holotype**
C. paurianus	CAL 1926 (KD22-009)	India: Uttarakhand	-	NG_242126	-	OQ914388	Holotype
C. paurianus	CAL 1927 (KD22-008)	India: Uttarakhand	-	OQ859920	-	OQ914389	Paratype
C. poikilochromus	**SOMF 30350**	**Bulgaria: Haskovo Prov.**	**OL774786**	**-**	**-**	**-**	**-**
C. poikilochromus	**K-M001441529 (AB B12-070)**	**Israel: Carmel Mount**	**PZ244182**	**-**	**-**	**-**	**-**

C. poikilochromus	**K-M001441522 (HAI A-063)**	**Israel: Lower Galilee**	**PZ244181**	**-**	**-**	**-**	**-**
C. poikilochromus	**K-M001441531 (AB B11-02)**	**Israel: Upper Galilee**	**PZ244183**	**PZ231927**	**-**	**-**	**-**
C. poikilochromus	**K-M001441521 (AB B12-085)**	**Israel: Upper Galilee**	**PZ244180**	**PZ231928**	**-**	**-**	**-**
C. poikilochromus	**IB1996585**	**Italy: Emilia-Romagna**	**PZ244176**	**PZ231929**	**-**	**-**	**Paratype**
C. poikilochromus	**VAL_Myco 1755 (IGB1667)**	**Spain: Valencian Com.**	**PZ244178**	**-**	**-**	**-**	**-**
C. poikilochromus	**VAL_Myco 1756 (IGB1673)**	**Spain: Valencian Com.**	**PZ244179**	**-**	**-**	**-**	**-**
C. poikilochromus	**VAL_Myco 1768 (IGB1422)**	**Spain: Valencian Com.**	**PZ244177**	**-**	**-**	**-**	**-**
C. poikilochromus	TUR-A 208926	Italy: Lombardia	MZ265177	MZ265192	MZ277233	MZ277222	-
C. poikilochromus	TUR-A 208927	Italy: Piemonte	MZ265178	MZ265193	-	MZ277223	-
C. poikilochromus	GS10070	Italy	KT157051	KT157060	KT157072	KT157068	-
C. poikilochromus	GS11008	Italy	KT157050	KT157059	KT157071	KT157067	-
C. poikilochromus	MG271	Italy	KT157048	KT157057	KT157070	-	-
C. poikilochromus	MG367	Italy	KT157049	KT157058	-	-	-
C. poikilochromus	TO HG 10091987	Italy	KT157047	KT157056	-	-	Epitype
C. poikilochromus	AQUI 7195	Italy	KT157052	KT157061	-	-	-
C. pulverulentus	**K-M001445829 (AB B18-391)**	**Hungary: Borsod–Abaúj–Zemplén Co.**	**PZ244190**	**-**	**-**	**-**	**-**
C. pulverulentus	**K-M001445822 (AB B18-392)**	**Hungary: Nógrád Co.**	**PZ244189**	**-**	**-**	**-**	**-**
C. pulverulentus	**GS10257**	**Italy: unknown**	**PZ244185**	**-**	**-**	**-**	**-**
C. pulverulentus	**GS10910**	**Italy: Sicily**	**PZ244186**	**-**	**-**	**-**	**-**
C. pulverulentus	**GS11186**	**Italy: Toscana**	**PZ244187**	**-**	**-**	**-**	**-**
C. pulverulentus	**PO-F2601**	**Portugal: Azores**	**PV461258***	**-**	**-**	**-**	**-**
C. pulverulentus	**K-M001445690 (AB B21-386)**	**UK: England**	**PZ244188**	**-**	**-**	**-**	**-**
C. pulverulentus	**MG1050**	**UK: England**	**PZ244191***	**-**	**-**	**-**	**-**
C. pulverulentus	**K-M001448123 (AB W5)**	**UK: Wales**	**PZ244184***	**-**	**-**	**-**	**-**
C. pulverulentus	RW109	Belgium	-	-	KT824046	KT824013	-
C. pulverulentus	PRM 944013	Czechia: Central Bohemia	LT714707	-	-	-	Epitype
C. pulverulentus	PRM 944014	Czechia: Central Bohemia	LT714705	-	-	-	-
C. pulverulentus	PRM 935997	Czechia: South Bohemia	LT714709	-	-	-	-
C. pulverulentus	PRM 944022	Czechia: Highland	LT714708	-	-	-	-
C. pulverulentus	PRM 944001	Czechia: Central Bohemia	LT714706	-	-	-	-
C. pulverulentus	MG126	Italy: Lombardy	KT157053	KT157062	-	-	-
C. pulverulentus	MG628	Italy: Lazio	KT157055	KT157064	KT157073	KY157069	-
C. pulverulentus	CA050916-04	Italy	HM347646	-	-	-	-

C. pulverulentus	MCVE 18188 (GS1826)	Italy: Emilia Romagna	JF907794	-	-	-	-
C. pulverulentus	TUR-A 209431	Italy: Sicily	MZ265184	-	-	-	-
C. pulverulentus	MG456	Portugal: Azores	KT157054	KT157063	-	-	-
C. pulverulentus	PRM 935923	Portugal: Madeira	LT714704	-	-	-	-
C. pulverulentus	clone 15892	Spain	MW282492	-	-	-	Environmental
C. pulverulentus	clone 13143	Spain	MW282441	-	-	-	Environmental
C. pulverulentus	clone 14181	Spain	MW282401	-	-	-	Environmental
C. pulverulentus	clone 12944	Spain	MW282397	-	-	-	Environmental
C. pulverulentus	A7	-	JX434685	-	-	-	-
C. pulverulentus	A21	-	JX434686	-	-	-	-
C. sinopulverulentus	**HMAS 266894**	**China: Shaanxi**	**PZ244192**	-	-	-	**Holotype**
C. sinopulverulentus	BJTC FM2319-A	China: Shanxi	NR_191306	NG_243401	OR659986	OR659937	Holotype of *C. flavocontextus*
C. sinopulverulentus	BJTC FM2319-B	China: Shanxi	-	OR655226	OR660025	OR659976	Paratype of *C. flavocontextus*
C. viscidiceps	HMJAU68168	China: Jilin	-	OR673995	OR683468	OR683479	Holotype
Cyanoboletus sp. 1	TN1601	Japan: Tokyo	LC832002	-	-	-	-
Cyanoboletus sp. 1	ASIS22672	South Korea?	KP004920	-	-	-	-
Cyanoboletus sp. 2	OR0322	Thailand	-	-	MH614722	MH614768	-
Cyanoboletus sp. 3	HKAS 59418	China	-	KT990570	KT990765	KT990403	-
Cyanoboletus sp. 3	HKAS 52639	China	-	KF112367	KF112195	KF112701	-
Cyanoboletus sp. 3	OR0257	China	-	-	MG212587	MG212629	-
Cyanoboletus sp. 3 (as Uncultured fungus)	1202_TKK_mateba	Japan: Kagoshima	LC806658	-	-	-	Environmental
Cyanoboletus sp. 3 (as *C. flavocontextus*)	LE F-344051	Vietnam	PP317924	PP313111	PP320320	-	-
Cyanoboletus sp. 3 (as *C. flavocontextus*)	LE F-344052	Vietnam	PP317923	-	PP320319	-	-
Cyanoboletus sp. 4	HKAS 90208-1	China	-	KT990571	KT990766	KT990404	-
Cyanoboletus sp. 4	HKAS 90208-2	China	-	-	KT990767	KT990405	-
Cyanoboletus sp. 5 (as *C.sinopulverulentus*)	HKAS 59609	China: Yunnan	-	KF112366	KF112193	KF112700	-
Cyanoboletus sp. 5 (as *C. sinopulverulentus*)	DC 16-51	India: Sikkim	-	MH684757	-	-	-
Cyanoboletus sp. 5	OR0961	Thailand	-	-	MH614724	MH614770	-
Cyanoboletus sp. 6	OR0491	China	-	-	MH614723	MH614769	-
Cyanoboletus sp. 7	HKAS 76850	China	-	KF112343	KF112187	KF112697	-
Lanmaoa angustispora	HKAS 74765	China	-	KF112322	KF112159	KF112680	-
L. angustispora	HKAS 74752	China	-	NG_059537	KM605154	KM605177	Holotype
L. asiatica	HKAS 63516	China	-	KT990584	KT990780	KT990419	-
L. asiatica	HKAS 63603	China	-	KM605143	KM605153	KM605176	-
L. flavorubra	NY775777	Costa Rica	-	NG_060283	KF112160	KF112681	Holotype
Rugiboletus brunneiporus	HKAS 83209	China	-	KM605134	KM605144	KM605168	Holotype
Rugiboletus sp.	M745	China	OR558330	OR554014	OR566420	OR566425	-

Table S3. Substitution models and partition schemes used in multi- and single-locus phylogenetic analyses.

Phylogenetic analysis	Marker	Partition	Nucleotide substitution model
Multi-locus	ITS	ITS1+ITS2	SYM + Γ
		5.8S	F81 + I
	LSU	-	GTR + Γ + I
	tef1-α	exon (1st cod. pos.)	HKY + I
		exon (2nd cod. pos.)	F81 + I
		exon (3rd cod. pos.)	GTR + Γ
		intron	HKY + Γ
	rpb2	exon (1st cod. pos.)	HKY + I
		exon (2nd cod. pos.)	F81 + I
		exon (3rd cod. pos.)	HKY + Γ
		intron	
	Scheme description used in multi-locus phylogenetic analysis: (ITS1+ITS2) (5.8S, rpb2_exon_2nd, tef1-α_exon_2nd) (LSU) (rpb2_exon_1st, tef1-α_exon_1st) (tef1-α_exon_3rd) (rpb2_exon_3rd, rpb2_intron, tef1-α_intron)		
Single-locus	ITS	ITS1+ITS2	SYM + Γ
		5.8S	JC + I
	Scheme for ITS: (ITS1+ITS2) (5.8S)		
	LSU	-	GTR + Γ + I
	tef1-α	exon (1st cod. pos.)	F81 + I
		exon (2nd cod. pos.)	JC + I
		exon (3rd cod. pos.)	GTR + Γ
		intron	K80 + Γ
	Scheme for tef1-α: (tef1-α_exon_1st) (tef1-α_exon_2nd) (tef1-α_exon_3rd) (tef1-α_intron)		
	rpb2	exon (1st cod. pos.)	K80 + I
		exon (2nd cod. pos.)	JC
		exon (3rd cod. pos.)	K80 + Γ
		exon (1st cod. pos.)	
	Scheme for rpb2: (rpb2_exon_1st) (rpb2_exon_2nd) (rpb2_exon_3rd, rpb2_intron)		
